# GABA(A) Receptor Activation Drives GABARAP–Nix Mediated Autophagy to Radiation-Sensitize Primary and Brain-Metastatic Lung Adenocarcinoma Tumors

**DOI:** 10.3390/cancers16183167

**Published:** 2024-09-15

**Authors:** Debanjan Bhattacharya, Riccardo Barrile, Donatien Kamdem Toukam, Vaibhavkumar S. Gawali, Laura Kallay, Taukir Ahmed, Hawley Brown, Sepideh Rezvanian, Aniruddha Karve, Pankaj B. Desai, Mario Medvedovic, Kyle Wang, Dan Ionascu, Nusrat Harun, Subrahmanya Vallabhapurapu, Chenran Wang, Xiaoyang Qi, Andrew M. Baschnagel, Joshua A. Kritzer, James M. Cook, Daniel A. Pomeranz Krummel, Soma Sengupta

**Affiliations:** 1 Department of Neurology and Rehabilitation Medicine, University of Cincinnati College of Medicine, Cincinnati, OH 45267, USA; bhattadj@ucmail.uc.edu (D.B.); kamdemde@ucmail.uc.edu (D.K.T.); vaibhav.gawali@hotmail.com (V.S.G.); kallaylm@ucmail.uc.edu (L.K.); 2 Department of Biomedical Engineering, University of Cincinnati, Cincinnati, OH 45221, USA; barrilro@ucmail.uc.edu; 3 Department of Chemistry and Biochemistry, Milwaukee Institute of Drug Discovery, University of Wisconsin, Milwaukee, WI 53211, USA; tahmed@uwm.edu (T.A.); rezvani3@uwm.edu (S.R.); capncook@uwm.edu (J.M.C.); 4 Department of Chemistry, Tufts University, Medford, MA 02144, USA; hawley.brown@tufts.edu (H.B.); joshua.kritzer@tufts.edu (J.A.K.); 5 Division of Pharmaceutical Sciences, University of Cincinnati College of Pharmacy, Cincinnati, OH 45229, USA; karveas@ucmail.uc.edu (A.K.); desaipb@ucmail.uc.edu (P.B.D.); 6 Department of Environmental & Public Health Sciences, University of Cincinnati, Cincinnati, OH 45267, USA; medvedm@ucmail.uc.edu; 7 Department of Radiation Oncology, University of Cincinnati College of Medicine, Cincinnati, OH 45219, USA; wang2kl@ucmail.uc.edu (K.W.); ionasctn@ucmail.uc.edu (D.I.); 8 Division of Biostatistics & Epidemiology, Cincinnati Children’s Hospital, Cincinnati, OH 45229, USA; nusratharun77@hotmail.com; 9 Division of Hematology and Oncology, Department of Internal Medicine, University of Cincinnati College of Medicine, Cincinnati, OH 45267, USA; subrahmaya@gmail.com (S.V.); qix@ucmail.uc.edu (X.Q.); 10 Department of Cancer Biology, University of Cincinnati College of Medicine, Cincinnati, OH 45267, USA; wang2cr@ucmail.uc.edu; 11 Department of Human Oncology, University of Wisconsin, Madison, WI 53792, USA; baschnagel@humonc.wisc.edu; 12 Department of Neurosurgery, University of North Carolina, Chapel Hill, NC 27599, USA; krummel@email.unc.edu; 13 Lineberger Comprehensive Cancer Center, University of North Carolina, Chapel Hill, NC 27599, USA; 14 Department of Neurology, University of North Carolina, Chapel Hill, NC 27517, USA

**Keywords:** lung cancer, brain metastasis, radiotherapy, autophagy, GABA receptor, GABARAP, Nix, p62, benzodiazepine

## Abstract

**Simple Summary:**

Non-small cell lung cancer (NSCLC) accounts for 80–85% of primary lung cancers. Radiation therapy is widely used to treat both primary NSCLC and lung cancer that has spread to the brain. However, radiotherapy responses are not durable and toxicity limits therapy. Therefore, there is an urgent need for new agents that can enhance the effectiveness of radiation therapy in lung cancer and reduce its toxicity. We find that AM-101, a benzodiazepine analog, enhances the effects of radiation and significantly improves the survival of mice with lung cancer brain-metastatic tumors. Additionally, AM-101 makes radiation treatment work better and slows down the growth of NSCLC subcutaneous xenograft tumors in mice. AM-101 activates the GABA(A) receptor in lung cancer cells, triggering selective autophagy by causing GABARAP to form multimers and stabilizing the mitochondrial receptor Nix. GABA(A) receptor activation may improve tumor control and allow for lower radiation doses, reducing toxicity.

**Abstract:**

In non-small cell lung cancer (NSCLC) treatment, radiotherapy responses are not durable and toxicity limits therapy. We find that AM-101, a synthetic benzodiazepine activator of GABA(A) receptor, impairs the viability and clonogenicity of both primary and brain-metastatic NSCLC cells. Employing a human-relevant ex vivo ‘chip’, AM-101 is as efficacious as docetaxel, a chemotherapeutic used with radiotherapy for advanced-stage NSCLC. In vivo, AM-101 potentiates radiation, including conferring a significant survival benefit to mice bearing NSCLC intracranial tumors generated using a patient-derived metastatic line. GABA(A) receptor activation stimulates a selective-autophagic response via the multimerization of GABA(A) receptor-associated protein, GABARAP, the stabilization of mitochondrial receptor Nix, and the utilization of ubiquitin-binding protein p62. A high-affinity peptide disrupting Nix binding to GABARAP inhibits AM-101 cytotoxicity. This supports a model of GABA(A) receptor activation driving a GABARAP–Nix multimerization axis that triggers autophagy. In patients receiving radiotherapy, GABA(A) receptor activation may improve tumor control while allowing radiation dose de-intensification to reduce toxicity.

## 1. Introduction

Lung cancer is the leading cause of cancer-associated mortality in the United States [1]. The most common lung cancer type, non-small cell lung cancer (NSCLC), comprises 80–85% of lung cancers and has an overall 5-year survival rate of 64%, but that drops to 37% once tumor cells invade the lymphatic system [1]. Most NSCLC patients will clinically present with metastatic disease, which is usually incurable. 

A highly challenging metastatic site to treat is the brain, which accounts for 30% of NSCLC metastatic cases [2]. However, post-mortem analysis of NSCLC patients suggests that the incidence of brain metastasis is significantly higher [3]. The standard of care for lung cancer brain metastasis includes surgical resection for large solitary lesions and stereotactic brain radiosurgery (SRS) in most other cases involving less than 10 lesions. Otherwise, whole brain radiation therapy (WBRT) is employed. However, both SRS and WBRT are associated with toxicity including radiation necrosis and neurocognitive effects, respectively. Managing potential toxicities and overcoming ensuing radiation resistance pose major challenges. While cranial radiotherapy is usually part of a multimodal treatment regimen for NSCLC brain metastasis, the median survival is only about 7–10 months [4,5,6]. To enhance radiation effectiveness, the plant alkaloid docetaxel (Taxotere) is commonly employed as a chemotherapeutic in conjunction with radiotherapy for advanced-stage NSCLC [7]. Docetaxel, however, is associated with significant co-morbidities, including weakened immunity and peripheral neuropathy. Furthermore, docetaxel is not effective for the treatment of brain metastasis, as it is not brain-penetrant. 

Significant attention has recently been focused on targeting autophagy as an anti-cancer stratagem with agents that inhibit or induce autophagy being explored. Agents that inhibit autophagy by interfering with the assembly of the double-layered autophagosomes include the immunosuppressive anti-parasitics like chloroquine and hydroxychloroquine [8]. Recently, cell-penetrant peptides have been engineered that inhibit autophagy more selectively by blocking autophagy-specific protein–protein interactions [9]. Agents that appear to induce autophagy include the anti-diabetic metformin and mTOR inhibitors everolimus, temsirolimus, and sirolimus (rapamycin). In support of an autophagic induction anti-cancer approach, recent reports suggest that the enhanced expression of key autophagy-associated proteins prolong patient survival [10,11,12]. The induction of autophagy by the plant-derived compound methyl jasmonate has been found to impart therapeutic effects in cultures of human NSCLC cells [13]. As well as molecules inducing autophagy, radiation is reported to enhance the expression of autophagy-associated proteins [14,15,16].

While the investigation of how stress induces autophagy is under intense study, leveraging the stress response vis a vis autophagy as an anti-cancer approach remains poorly explored. For example, the induction of autophagy is often elicited in response to mitochondrial stress [17,18]. Mediating the stress response are membrane-associated proteins that signal to alter a cellular metabolic state. Prominent membrane-associated proteins sensing environmental cues are the ligand-gated ion channels, which represent a significant pharmacologic target for the remediation of neurological disorders but remain poorly explored as therapeutic vulnerabilities in cancer. Amongst ion channels, the Type-A GABA receptors are a principal target. GABA(A) receptors function as the major inhibitory neurotransmitter receptors and hyperpolarize post-synaptic mature neurons in response to the binding of its agonist and metabolite, GABA. We and others have reported on the enhanced expression of GABA(A) receptor subunits in disparate cancers [19,20] and shown that GABA(A) receptors intrinsic to cancer cells are functional, i.e., GABA responsive. Importantly, GABA(A) receptors in cancer cells are depolarizing [21], which reflects an efflux of chloride anions like the embryonic receptor. The presence of GABA(A) receptors in cancer cells and its depolarizing effect when ‘activated’, in contrast to the adult receptor in non-cancer cells, prompted us to investigate the receptor as a therapeutic vulnerability to cancer cells [20]. This led to the identification of a class of brain-penetrant molecules that activate GABA(A) receptors and impair the viability of cancer cells as well as mediate tumor control in mouse models of these cancers. We also reported that a member of this class of activators, AM-101, potentiates radiation in a mouse melanoma tumor model [22]. AM-101 was previously named QH-II-066.

The study of AM-101’s mode of action revealed that it depolarizes the mitochondrial transmembrane potential of cancer cells as well as activating the intrinsic (mitochondrial) apoptotic pathway [21]. Mitochondrial dysregulation also induces lysosomal-dependent autophagy selectively in cancer cells [23,24] and, thus, a potential role for autophagy in AM-101’s mode of action. In support of such a role, the protein GABARAP contributes to both GABA(A) receptor function and autophagy. GABARAP is reported to mediate GABA(A) receptor subunit transport to the extracellular membrane, where it aids assembly as well as the multimerization of the receptor [25,26,27]. The multimerization of GABA(A) receptors is proposed to create a strong, localized distribution of charge and enhanced activity, as observed experimentally [26]. Interestingly, it is theorized that the binding of GABARAP to GABA(A) receptors would also enhance chloride anion conductance [28]. During autophagy, GABARAP is reported to multimerize and colocalize with the autophagosome-associated protein LC3-II, and this interaction serves to nucleate autophagosome assembly and facilitate the fusion of autophagosomes with lysosomes for cargo degradation [26,27,29,30]. GABARAP dimers also complex with dimers of the outer mitochondrial membrane protein BNIP3L/NIX or Nix, which is a crucial molecule in the selective autophagic response for the removal of stressed or damaged mitochondria [29]. These findings suggest that GABARAP may be a key intermediary molecule in regulating autophagy in response to ionic perturbation as mediated by GABA(A) receptors. 

Herein, we have evaluated GABA(A) receptor activation as an approach in conjunction with radiation to treat both primary and metastatic brain NSCLC. We find that the GABA(A) receptor activator AM-101 is as effective as docetaxel. In both heterotopic and intracranial NSCLC mouse models, the administration of AM-101 plus radiation leads to effective tumor control. In mice with metastatic intracranial tumors, this combination also extends survival. Mechanistically, AM-101 acts to drive a selective autophagic response that is dependent on the GABARAP–Nix complex formation. In addition, key proteins for the assembly of autophagosomes show enhanced expression when radiation and AM-101 are combined. AM-101 passes through the blood–brain barrier and enters lungs rapidly and is non-toxic in primates [31]. Activating GABA(A) receptors represents a new paradigm to treat lung cancer, most significantly brain metastatic. We present a new class of small molecule brain-penetrant autophagy inducers that conceptually support this strategy.

## 2. Materials and Methods

### 2.1. Cell Lines and Maintenance

Human non-small cell lung carcinoma lines A549, H1792, H460, and H1703 were purchased from the American Type Culture Collection (ATCC). UW-lung-16 is a lung adenocarcinoma brain-metastatic patient-derived cell line [32]. H1792, H460, and H1703 were grown in RPMI-1640 (Roswell Park Memorial Institute-1640) Medium (Gibco, Thermo Fisher Scientific, Waltham, MA, USA). A549 was grown in Dulbecco’s Modified Eagle Medium (DMEM) (Corning, Somerville, MA, USA). UW-lung-16 was grown in DMEM with glucose, L-glutamine, and sodium pyruvate. Media for all lines was supplemented with 10% (*v*/*v*) fetal bovine serum (FBS) (Corning) and 100 U/mL Penicillin–Streptomycin (Sigma, St. Louis, MI, USA). Lines were grown at 37 °C, 5% (*v*/*v*) CO_2_. BEAS-2B cells, which are normal-immortalized human bronchial epithelial cells, were purchased from ATCC, USA. BEAS-2B cells were grown in airway epithelial cell basal medium (ATCC, Manassas, FL, USA) supplemented with bronchial epithelial cell growth kit (ATCC). Ten percent fetal bovine serum (Corning) was added to the complete airway epithelial cell growth media. Both primary human NSCLC cell cultures and patient-derived UW-lung-16 cell lines were tested regularly and were negative for mycoplasma contamination.

### 2.2. Reverse Transcriptase PCR

Total RNA from confluent cells was isolated using Trizol reagent (Thermo Fisher Scientific, Waltham, MA, USA) and stored at −80 °C. First-strand cDNA was prepared from 1 µg total RNA using the ProtoScript II First Strand cDNA synthesis kit (New England Biolabs, Ipswich, MA, USA). PCR amplification for *GABRA5* mRNA (primers: forward, CTTCTCGGCGCTGATAGAGT; reverse, CGCTTTTTCTTGATCTTGGC) employed an initial denaturing step (95 °C, 5 min) followed by thirty-five cycles (95 °C, 30 s; 59 °C, 40 s; 72 °C, 40 s) and a final extension (72 °C, for 7 min). We used Platinum PCR SuperMix High Fidelity (Thermo Fisher Scientific) for the PCR amplification reactions. PCR amplification for *GAPDH* mRNA (forward, TGAAGGTCGGAGTCAACGGATTTGGT; reverse, CATGTGGGCCATGAGGTCCACCAC) employed an initial denaturing step (95 °C, 5 min) followed by thirty-two cycles (95 °C, 30 s; 57 °C, 40 s; 72 °C, 40 s) and a final extension at 72 °C for 7 min (Platinum^®^ PCR SuperMix High Fidelity, Invitrogen by Thermo Fisher Scientific). PCR amplification for *GABRA3* mRNA (primers: forward, TCGGTCTCTCCAAGTTTGTGC; reverse, TTCCGTTGTCCACCAATCTGA) employed an initial denaturing step (95 °C, 5 min) followed by thirty-two cycles (95 °C, 30 s; 57 °C, 40 s; 72 °C, 40 s) and a final extension at 72 °C for 7 min. PCR products were run on 1.5% ethidium bromide-stained agarose gel at 90 V using a mini horizontal electrophoresis system (Bio-Rad mini-sub cell GT cell, Bio-Rad, Hercules, CA, USA). Gel images were acquired using a Bio-Rad Gel Doc XR+ Imaging System. 

### 2.3. Immunohistochemistry

Patient lung adenocarcinoma tumor tissue (primary and matched brain metastatic) was obtained from the CLIA-certified University of Cincinnati Histopathology Core Laboratory under an approved IRB. Tissue (fixed in 4% paraformaldehyde/PBS and paraffin embedded) was obtained as 5 µm sections on glass slides, which were then deparaffinized and antigen retrieved by heat-induced epitope retrieval (HIER) using citrate buffer (low pH 6.0) for 15 min. Slides were incubated with 2.5% normal horse serum (supplied with Anti-Rabbit IgG Polymer PLUS kit, Vector Laboratories, Newark, CA, USA) for 20 min and then with GABRA5 and GABRA3 primary Ab (1:150 dilution) depending on the experiment for 15 min at room temperature. Polymer-based HRP-conjugated secondary Ab was incubated for 8 min at room temperature. BOND polymer refine detection kit (Leica Biosystems) was used for the chromogenic staining.

### 2.4. AM-101 and Pen3-ortho Stapled-Peptide Preparation

AM-101 was synthesized as described [33,34]. For in vitro and ex vivo studies, AM-101 was kept lyophilized at room temperature and solubilized in dimethyl sulfoxide (DMSO, 0.125%) prior to use. For mouse studies, AM-101 was solubilized in a co-solvent formulation (propylene glycol (40%); ethanol (10%); benzyl alcohol (2%); benzoic acid (2%); sodium benzoate (2%)) [35]. AM-101 (5 mg) was dissolved in 0.1 mL of ethanol (10%) at room temperature; then other components added with mixing, followed by 30 s vortex mixing. Pen3-*ortho* was synthesized as described [9].

### 2.5. Electrophysiology

Functional characterization employed a Port-a-Patch single-cell automated patch-clamp electrophysiology instrument (Nanion Technologies, München, Germany). Recording solutions (Nanion Technologies) were as follows: external solution, 140 mM NaCl; 4 mM KCl; 1 mM MgCl_2_; 2 mM CaCl_2_; 10 mM HEPES; 5 mM D-Glucose; high Ca^2+^ seal enhancer solution, 130 mM NaCl; 4 mM KCl; 1 mM MgCl_2_; 10 mM CaCl_2_; 10 mM HEPES; 5 mM D-Glucose; internal solution, 110 mM KF; 10 mM NaCl; 10 mM KCl; 10 mM EGTA; 10 mM HEPES, pH 7.2 adjusted using KOH. 

To increase the current amplitude in single-cell recordings, GABA and AM-101 were dissolved in a high-sodium-containing external solution (161 mM NaCl; 3 mM KCl; 1 mM MgCl_2_; 1.5 mM CaCl_2_; 10 mM HEPES; 6 mM D-Glucose). Whole-cell recordings were performed on cells (held at −80 mV) using a gap-free protocol under continuous perfusion of external solutions and drug applications. GABA (1 µM) and AM-101 (4 µM) were applied briefly for 5 s to record the current potentiation. Data acquisition was done using HEKA amplifier EPC 10 USB (Multi channel systems MCS GmbH, Reutlingen, Germany). Data were low-pass filtered at 1 kHz and digitalized at 100 kHz. Data analysis was performed by computing the maximum current amplitude using Nest-o-Patch version 2.1.6 software (Open Source).

### 2.6. Mitochondrial Depolarization

AM-101 was diluted to 4 µM in RPMI-1640 culture media. Carbonyl cyanide 4-(trifluoromethoxy) phenylhydrazone (FCCP) (Sigma) was diluted to 20 µM in culture medium. Tetramethylrhodamine ethyl ester perchlorate (TMRE) (Sigma) was diluted to 400 nM in cell culture medium. H1792 cells were grown in culture to 75–90% confluency. Cells (5 × 10^5^ cells/mL) were harvested and resuspended in media. Cell suspension (200 µL) was dispensed and AM-101 or FCCP (200 µL) added to final concentrations of 2 µM and 10 µM, respectively. Solution was briefly vortexed and incubated for 10 min. TMRE (40 µL of 400 nM stock) was added (final concentration 10 nM) and vortexed, and the sample reading was acquired using a BD LSR Fortessa (Beckton Dickinson, Franklin Lakes, NJ, USA). Data were analyzed using FlowJo v10.8.1 software (FlowJo, LLC, Ashland, OR, USA).

### 2.7. Preparation of Lung Cancer Chips

Human Alveolar Epithelial Cells (HPAECs) (Cell Biologics, Chicago, IL, USA) were cultured on a Matrigel/collagen-coated T25 flask for 4–7 days in serum-free Small Airway Epithelial Cell Growth Medium (SAGM) (Lonza, Basel, Switzerland) and used without further passage. Human Lung-Microvascular Endothelial Cells (HMVEC-Ls) (Lonza) were grown on a collagen-I-coated flask (T75) in microvascular cell culture medium (Lonza) and used between passages 4 and 5.

Lung chips are composed of transparent material made of a tetrafluoroethylene-propylene (FEPM) elastomer containing two parallel microchannels separated by a thin collagen vitrigel membrane, which was previously described and characterized [36]. In contrast to previous organ-on-chip models of the lung [37,38,39] we used a PDMS-free design. The chips were a kind gift of Naoki Matsuoka (AGC Inc., Tokyo, Japan). Before the experiment, both microchannels were chemically functionalized using 3-aminopropyl-trimethoxysilane (APTMES) (Sigma) to covalently bind extracellular matrix (ECM) proteins before seeding human cells, as previously described [40]. The apical or epithelial channel (1 × 1 mm) was coated with a mix of human placenta collagen IV (200 μg/mL) (Sigma-Aldrich), human placenta laminin (10 μg/mL) (Sigma-Aldrich), and human plasma fibronectin (30 μg/mL) (Corning), all resuspended in Hanks’ Balanced Salt Solution (HBSS) (Sigma-Aldrich). The bottom or vascular channel (200 μm × 1 mm) was coated with human placenta collagen type IV (200 μg/mL) (Sigma-Aldrich) and human plasma fibronectin (30 μg/mL) (Corning). Chips were incubated overnight at 37 °C to complete the surface coating. The next day, HPAECs were seeded in the epithelial channel at a density of ~0.8 × 10^6^ cells/mL in SAGM, and chips were incubated at 37 °C. Three hours after cell seeding, both epithelial and vascular channels were rinsed 2X with 100 μL SAGM to remove unattached cells. Medium was then replaced with SAGM supplemented with keratinocyte growth factor (10 ng/mL), isobutylmethylxanthine (100 μM), 8-bromo-cyclicAMP (100 μM), and dexamethasone (200 μM) (KIAD). The medium in the epithelial channel (SAGM + KIAD) was replaced once a day for 6 days. Three days after epithelial cell seeding, the vascular compartment was seeded with HMVEC-L at a density of ~8 × 10^6^ cells/mL in microvascular cell culture medium (EGM-2 MV) (Lonza), and chips were then incubated at 37 °C. One hour after endothelial cell seeding, the vascular channel was rinsed 2X with cell culture medium to remove free cells. The vascular medium was then refreshed once a day with EGM-2 MV for 3 days. One day after endothelial cell seeding, H1792-GFP cancer cells were seeded on the epithelial compartment at a density of ~3.5 × 10^5^ cells/mL, as previously described [41]. The epithelial channel was rinsed with fresh cell culture medium 3–5 h after seeding of cancer cells, and the air–liquid interface (ALI) was established the next day via pipetting 50 μL of air in the epithelial channel. Chips were then connected to flow using a syringe pump and perfused with a modified endothelial cell culture medium (ALI medium) made with Medium 199 (ThermoFisher) supplemented with 2% HyClone FetalClone II Serum (GE Healthcare Life Sciences, Chicago, IL, USA), 10 ng/mL Epidermal Growth Factor (PromoCell, Heidelberg, Germany), 10 ng/mL Keratinocyte Growth Factor (PromoCell), 0.25 ng/mL Vascular Endothelial Growth (PromoCell), 1 μg/mL Hydrocortisone (Sigma), 1 U/mL Heparin (Sigma), 50 μM 8-Bromoadenosine 3′,5′-cyclic monophosphate (Sigma), GlutaMAX (Thermo-Fisher Scientific), 20 nM Dexamethasone (Sigma), and Penicillin–Streptomycin (GIBCO).

### 2.8. Imaging of Lung Cancer Chips

Fluorescence images were captured using an Olympus IX83 microscope equipped with a 20X apochromatic objective. To generate growth curves of cancer cells cultured on-chip, consistent exposure times were maintained for H1792 GFP+ cells across all time points. A minimum of *n* = 3 images per chip were acquired from *n* = 3 chips, followed by background signal removal and analysis using Fiji version 2.1.0 (Image J). Sequential signal thresholding was applied, and the percentage of area covered by the signal was measured. Similarly, the impact of doc on-chip was assessed using background subtraction and signal thresholding, followed by particle analysis using a Fiji plugin to estimate the number of GFP+ cells per field of view in each testing condition. The choice of particle analysis over percentage of area covered by the signal was based on its ability to provide discrete values, resulting in a more sensitive measurement.

### 2.9. Immunoblotting

Tumor tissue (mouse heterotopic xenograft or intracranial xenograft) was harvested, minced on ice, and lysed in ice-cold RIPA buffer (Thermo Fisher Scientific) supplemented with protease and phosphatase inhibitor cocktail. DNA was sheared by sonication using a sonicator. For adherent human cancer cells, after an initial wash with sterile cold PBS, the cells were harvested from the wells of 6-well culture plates using cold trypsin-EDTA. The cells were then washed with PBS once and collected by centrifugation in sterile microcentrifuge tubes. Cells in pellets were lysed in ice-cold RIPA buffer (Thermo Fisher Scientific) supplemented with protease and phosphatase inhibitor cocktail. DNA was sheared by sonication using a sonicator as described earlier. Lysates (from cells or tissues) were kept on ice for 30 min, then centrifuged at 13,500 × *g* for 10 min at 4 °C. Protein concentration in the supernatant was measured using the Bradford assay with Bio-Rad Protein Assay dye reagent. After the protein estimation, lysates containing 30 μg of protein were mixed 1:1 (by volume) with 2X Laemmli sample buffer containing beta-mercaptoethanol and heated for 5 min, 95° C. Equal amount of protein (30 μg) sample was loaded in each well of 4–20% gradient polyacrylamide gels (Bio-Rad), resolved by SDS-PAGE, and then transferred to nitrocellulose membranes (Bio-Rad) for 2 h at 100 V in tris-glycine transfer buffer containing 20% methanol. Membranes were blocked at room temperature in 1X TBST blocking buffer (TBS with 0.1% Tween-20 and 5% non-fat dry milk) for 1 h with gentle agitation, followed by overnight incubation with primary Ab and gentle shaking at 4° C. The primary antibodies were diluted as follows: GABRA5 (1:1000, Aviva, London, UK); β-actin (1:1000, Cell Signaling Technology, Danvers, MA, USA); GAPDH (1:1000, Cell Signaling Technology); GABARAP (1:1000, Cell Signaling Technology); ATG7 (1:1000, Cell Signaling Technology); ATG12 (1:1000, Cell Signaling Technology); NIX (1:1000, Cell Signaling Technology); Beclin-1 (1:1000, Cell Signaling Technology); p62/SQSTM1 (1:1000 in 5% milk in TBST, Cell Signaling Technology). Immunodetection was performed with anti-rabbit horseradish-peroxidase-conjugated secondary antibody (1:10000, Cell Signaling Technology). Post-primary Ab incubation, membranes were washed (3X for 10 min in TBST at room temperature), probed with rabbit-HRP-tagged secondary antibody (1:3000, Cell Signaling Technology), and processed for chemiluminescence detection using ECL detection substrate (Super Signal West Pico Plus Chemiluminescent substrate by Thermo Scientific, USA). Chemiluminescence images were acquired using the ChemiDoc Touch Imaging System (Bio-Rad, USA). 

### 2.10. Immunofluorescence Staining and Confocal Imaging

Cells were seeded on sterile glass cover slips and grown overnight at 37 °C. Cells were rinsed with cold PBS and fixed in 4% paraformaldehyde (30 min, room temperature). Fixed cells were rinsed in PBS (3 times for 5 min each at room temperature) and permeabilized (15 min in PBS containing 0.1% Triton X-100). Cells were washed in PBS (3 times for 5 min each wash at room temperature) and incubated with blocking buffer (PBS containing 0.3% Triton X-100 and 3% BSA) for 1 h at room temperature with gentle shaking. The blocking solution was aspirated, and the cells were washed with ice-cold PBS. The cells were then incubated overnight at 4 °C with primary antibody (LC3B or Nix depending on the experiment) diluted 1:200 in sterile 0.5% BSA in PBS on a coverslip placed on a glass slide, and kept inside a humidified 10 cm plastic Petri dish [42]. Cells were washed (3X for 5 min in PBS) and incubated with fluorophore-conjugated secondary Ab goat anti-rabbit Alexa Fluor 594 (Abcam, Cambridge, UK) in 2% normal donkey serum at room temperature for 1 h in dark. Cells were washed (3X for 5 min in PBS) under low light and mounted on a glass slide in Vectashield Antifade Mounting Medium with DAPI (Vector Laboratories). The following Ab’s were used (1:200 dilution): rabbit anti-human LC3B (Cell Signaling Technology) and rabbit Nix (Cell Signaling Technology). Cells were also prepared as negative control where the primary antibody was omitted. Slides were imaged on a Zeiss LSM 710 laser scanning confocal microscope with a Zeiss Axio Observer Z1 stand and a Zeiss Plan-Apochromate objective (63x/1.4 Oil DIC). The appropriate lasers and emission filters for the respective fluorophores were used during image acquisition. Images were analyzed using the Fiji (version 2.1.0) package of ImageJ2 [43].

### 2.11. Clonogenic and In Vitro Viability Assays

For the clonogenic assay, a fixed number of cells were seeded (200 cells per well, H1792; 150 cells per well, UW-lung-16) in 4 wells of a 6-well cell culture plate, grown overnight in humidified conditions at 37 °C, and treated with AM-101 or DSMO for 1 h before irradiation. Each treatment group had four replicates. Once colonies averaged 40 or more cells (14–18 days in control wells), media were removed, and cells were washed with sterile PBS. Cells were fixed for 30 min with methanol–acetone (3:1 by volume) and stained with 1% (*w*/*v*) crystal violet in methanol for 30 min. Stained colonies were gently washed with distilled water, dried, and photographed on a white background. Colonies of 40 or more cells counted in radiation groups were irradiated one day after seeding with a single fraction X-ray source, XenX irradiator (Xstrahl Ltd., Suwanee, GA, USA), at room temperature. The XenX irradiator uses a collimator to deliver a uniform dose with a posterior–anterior (PA) beam focused on the tops of the wells. For the control group (non-irradiated and untreated plates), media were replaced with fresh media after 72 h. Colonies were allowed to grow for 14 days in the H1792 cell group and for 18 days in the UW-lung-16 cell group following irradiation. Media were removed and colonies washed with PBS, fixed, and stained. The clonogenic survival curve for each condition was fitted to a linear quadratic model according to least squares fit, weighted to minimize the relative distances squared, and was compared using the extra sum-of-squares F test. Each point represents the mean surviving fraction calculated from four replicates, and error bars represent the standard error (SE). Data are represented as mean ± SE of colonies using bar graphs and were analyzed using GraphPad Prism 8. *p*-values were calculated using one-way ANOVA with Tukey’s multiple comparison test (GraphPad Prism 8). 

Cell proliferation viability studies employed the Cell Titer 96 Aqueous One Solution Assay (Promega, Madison, WI, USA), and the assay was performed as described in an earlier study [44]. IC_50_ values were determined using the ‘[Inhibitor] versus normalized response’ nonlinear regression function and the log of inhibitor concentration in Prism 8 software (version 8.3.0 for macOS).

### 2.12. Irradiation of NSCLC Cells

Human primary H1792 cells or patient-derived brain-metastatic UW-lung-16 cells were seeded on 6 cm tissue culture dishes and maintained in respective medium supplemented with FBS and antibiotics. Cells at 70% confluency were irradiated at respective doses of radiation, using a XenX irradiator (Xstrahl Ltd.) at room temperature. Cells were harvested at indicated time points and either lysed for immunoblotting or washed and fixed with 4% paraformaldehyde for confocal immunofluorescence microscopy.

### 2.13. In Vitro Cell Survival Assay with Stapled-Peptide and AM-101

H1792 cells were plated with phenol-red free RPMI-1640 media (2500 cells per well in 100 μL media) and grown overnight. The next day, media were removed, fresh 100 μL phenol-red free RPMI media added, and the following treatment groups setup: (1) Pen3-*ortho* group (15 μM); (2) Pen3-*ortho* (25 μM); (3) AM-101 group (3 μM); (4) combination group at AM-101 (3 μM) and Pen3-*ortho* (15 μM); (5) combination group at AM-101 (3 μM) and Pen3-*ortho* (25 μM); (6) DMSO control with no drug added; (7) control with no cells added, i.e., media alone. Cells were then incubated at 37 °C, 5% CO_2_ for 48 h. Following this, 20 μL of diluted MTS reagent was added, cells incubated at 37 °C for 1 h, and absorbance (490 nm) was measured using a microplate reader (Molecular Devices, Wokingham, UK). The mean of the absorbance of media-only samples was subtracted from control and test group samples. The percentage of inhibition in each group was calculated by the formula (C − T)/C × 100%, where ‘C’ is the mean absorbance reading for the control group, and ‘T’ is the mean absorbance reading for each treated group. The percentage of survival was calculated (GraphPad Prism 8.0.1) by subtracting the percentage of inhibition of each group from 100% and expressed as mean ± SEM. Student’s *t* test (paired) for two groups were used for statistical comparison. A *p* < 0.05 is considered significant.

### 2.14. Mouse Experiments

#### 2.14.1. Subcutaneous Xenograft Studies

Mice were housed in pathogen-free rooms, and clinical health was evaluated weekly by veterinary staff (University of Cincinnati LAMS). All animal studies were conducted in accordance with IACUC approval (University of Cincinnati). For subcutaneous xenograft tumor growth delay experiments, NOD-SCID gamma (NSG) mice were used (Charles River Laboratories, Wilmington, MA, USA). For intracranial xenograft tumor experiments, athymic nude mice were used (Envigo, Indianapolis, IN, USA). For mice receiving radiation, tumors were irradiated using an XenX small animal irradiator (Xtrahl, Inc., Sugar Hill, GA, USA). 

For heterotopic xenograft experiments, H1792 cells (0.5 × 10^6^ cells per mouse) grown in RPMI medium were washed in cold PBS, mixed with Matrigel (25%), and injected subcutaneously into left and right flanks above the hind limbs of 6- to 8-week-old female mice. When subcutaneous tumors were palpable (35 days; ~100 mm^3^) the following treatments groups were initiated: (1) vehicle; (2) radiation; (3) AM-101 ± radiation; and (4) docetaxel ± radiation. In vehicle mice, vehicle was injected i.p. for 7 days. In radiation mice, left-side flank tumors were irradiated with a single 5 Gy dose. In radiation plus drug mice, AM-101 (2.5 mg/kg body weight) or docetaxel (8 mg/kg body weight) was injected i.p. 1 h prior to irradiation with a single 5 Gy dose. AM-101 or docetaxel was subsequently injected i.p. for 7 days. Following the end of the treatment, tumor volume and body measurements were taken for growth delay studies. Mice were euthanized using CO_2_ euthanasia when the experimental endpoints according to the animal care guidelines were reached, and subcutaneous tumors were excised and weighed and preserved at appropriate conditions for protein and IHC studies. The day of tumor cell implantation in mice was assigned as day zero in the timeline of the experiment. Mouse tumors were measured by Vernier calipers. Tumor volume was calculated using the formula: (π/6) × (*l* × *h*^2^), where *l* and *h* are the large diameter and small diameter of the tumor taken perpendicular to each other. 

#### 2.14.2. Intracranial Xenograft Studies

For intracranial implant experiments, UW-lung-16 PDX cells stably transduced with luciferase were harvested from culture and washed in sterile PBS and suspended in cold sterile PBS. Viable cell numbers were calculated using Trypan blue staining and adjusted for intracranial implantation. Mice eyes were protected using an Artificial Tears solution. Mice were put under isoflurane anesthesia using an inhalation anesthesia machine (VetEquip, Pleasanton, CA, USA). Analgesic buprenorphine was administered subcutaneously to each mouse before starting the procedure. The skin on the skull of the mouse to be injected was disinfected using isopropanol and chlorohexidine gluconate, and a small incision was made on the skin to expose the skull. From the bregma, the syringe was moved to the appropriate AP and lateral coordinates, and a small hole was drilled using a 26-gauze needle. Using a Hamilton syringe, 2 × 10^5^ cells suspended in 3 µL sterile PBS were implanted by stereotactic injection into the right striatum of each 6-week-old athymic nude mouse with the following stereotactic landmarks: 2 mm right lateral and 0.5 mm frontal to the bregma at 3 mm depth. The stereotactic injection in each mouse occurred over 5–6 min. Following stereotactic injection, bone wax was applied at the hole in the skull and scalp closed with sterile staples. Mice were kept on a heating pad during the procedure and to recover from anesthesia. The calculated median survival of mice with implanted brain-metastatic tumors and receiving no treatment was 31 days after implantation. Successful tumor engraftment was ultimately confirmed by bioluminescence imaging (using an Fx Pro preclinical imager, Bruker, Billerica, MA, USA) 8 days after implantation. Eight days after implantation, animals were randomly assigned to three experimental groups: (1) vehicle only; (2) radiation plus vehicle; (3) AM-101 plus radiation (N = 7 mice per group). AM-101 was administered at a dose of 5 mg/kg per mouse by i.p. injection daily for 7 days. Whole-brain radiation was administered 2.5 Gy daily fraction for 5 days using XenX small animal irradiator and a supplied mouse gantry (Xtrahl, Inc). AM-101 was given 1 h before radiation treatment [5]. We chose the fractionated radiation dose based on radiation-sensitizing experiments that previously employed this UW-lung-16 mouse model [5]. The day of tumor implantation was assigned as day zero. Mouse brain tumor growth and spinal lesion(s) (if any) were evaluated by luciferase-based BLI on days 8, 18, 26, 33, and 56. A Bruker multi-spectral Fx Pro preclinical imager was used for luciferase imaging. For luciferase imaging, each mouse received intraperitoneal injections of freshly prepared sterile a D-Luciferin, potassium salt solution (15 mg/mL) in 0.9% saline solution. Each mouse received a D-luciferin injection based on their body weight (10 µL of stock solution per 10 g of body weight). Mice were imaged 10 min after D-luciferin injections. Mouse body weight was also measured over time, and mice were regularly monitored for symptoms. Mice were euthanized once experimental endpoints were reached. Mouse brain tumor tissue from the cerebral cortex was dissected and processed for further analysis like protein extraction, etc.

To measure mean flux intensity, the bioluminescence signal was quantified by using flux (photons/s/mm^2^) in a region of interest (ROI), drawn manually to outline the whole area of the signal from the mouse brain tumor. Mean flux intensity was measured using Carestream Molecular Imaging Software, version 5.3.4.

### 2.15. Data Analysis and Statistics

Data for cell survival experiments are expressed as mean ± SEM. Data analysis employed GraphPad Prism 8.0.1 software. All figures were assembled with Microsoft Office Professional Plus 2019. Statistical differences between two groups were determined using a two-tailed unpaired Student’s *t*-test. To account for multiple comparisons, the c was applied, adjusting the *p*-value threshold accordingly. A statistician was involved in overseeing the statistical analysis. One-way ANOVA with Turkey’s post-hoc test for multiple group comparisons was performed with the assumption of Gaussian distribution of residuals. Kaplan–Meier curves were used for calculating survival for the intracranial experiments, and statistical significance was determined by the log-rank test. Prism 8.0 software was used for all statistical calculations. A *p* < 0.05 is considered significant. 

## 3. Results

### 3.1. Lung Adenocarcinomas Express GABA(A) Receptor Subunits

**Figure 1:**
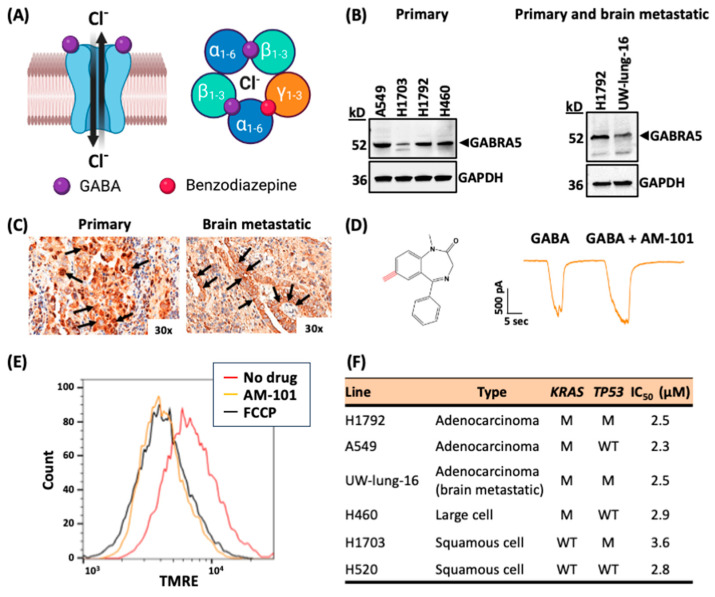
Activation of GABA(A) receptors triggers cell depolarization and death. (**A**) Type-A GABA (GABA(A)) receptors are ligand-gated chloride anion channels. Left, GABA(A) receptors move chloride anions (Cl^−^) out of the cell during embryonic stages of development but into the cell in mature or developed stages and are thereby depolarizing or hyperpolarizing, respectively. Right, GABA(A) receptors form hetero-pentameric structures with an α2β2γ1 stoichiometry. Two molecules of GABA (purple spheres) bind at the α-β interfaces to ‘activate’ receptor function (chloride anion transport). Commonly, one molecule of benzodiazepine (red sphere) binds at the α-γ interface to enhance flow of chloride anions. Created with BioRender.com (**B**) Left, GABRA5 or α5 protein in NSCLC patient-derived primary cell lines representing three histological subtypes (adenocarcinoma, A549, H1792; squamous cell, H1703; large cell, H460). Right, GABRA5 or α5 protein expression in patient-derived lung adenocarcinoma brain-metastatic cell line (UW-lung-16), and primary human lung adenocarcinoma cell line (H1792). The presence of protein confirmed by Western blotting of SDS (4–15% gradient) gels. GAPDH is used as a loading control. Cropped gel lanes from original blots, see Figure S8. (**C**) NSCLC primary (left) and brain-metastatic (right) patient tumor tissue from the same patient (or matched) stains for GABRA5 or α5 protein, as shown by immunohistochemistry staining at 30× magnification. Arrows show GABRA5 staining in large tumor cells within primary and brain-metastatic lung adenocarcinoma tissue sections. (**D**) AM-101 (QH-II-066) (274.32 g/mol) is a benzodiazepine analog (left). A representative single cell patch clamp electrophysiology trace of patient-derived adenocarcinoma lung cell line H1792 (right). Cells are responsive to GABA or electro-physiologically functional. Perfusion of cells with AM-101 plus GABA elicits an enhanced response, indicating that GABA(A) receptors are benzodiazepine-responsive or ‘activated’. Representative raw current trace recording with the following parameters: GABA, 1 µM; AM-101, 4 µM. (**E**) Lung adenocarcinoma (H1792) cells incubated with AM-101 are depolarized, as assessed by the TMRE assay and Fluorescence-Activated Cell Sorting (FACS) analysis. Shown is the degrees of depolarization relative to DMSO-alone treatment and FCCP, which provide negative and positive controls in this experiment, respectively. Parameters: AM-101, 2 µM; FCCP, 10 µM. (**F**) Half-maximal inhibitory concentration (IC_50_) values of patient-derived lung primary and brain-metastatic lines representative of three histological lung cancer subtypes, as measured using a viability (MTS) assay and AM-101. Indicated is the KRAS and TP53 mutational status of lines, where M represents mutant, and WT represents wild-type.

GABA(A) receptors are ligand-gated anion channels that move chloride anions across the extracellular membrane in response to the binding of its natural agonist and metabolite, GABA (Figure 1A). GABA(A) receptors form pentameric assemblies from a combination of the translation product(s) of nineteen possible GABR genes [31]. The canonical hetero-pentameric receptor has an α2-β2-γ stoichiometry with subunits primarily sampled from six α (GABRA1-6), three β (GABRB1-3), and three γ’s (GABRG1-3) (Figure 1A). The analysis of the TCGA cohort of primary lung cancer patient tumors across NSCLC-subtype squamous cell carcinoma and adenocarcinoma shows the expression of GABR genes. All squamous cell carcinoma tumors express a distinct set of GABR genes that are not expressed in normal tissue, including GABRE and GABRA3 (Figure S1A), while adenocarcinomas show a distinct gene expression signature for a subset of patients or, otherwise, the expression of GABR genes broadly (Figure S1B). Western blots with a panel of human non-small cell lung cancer primary tumor cell lines (H1792, A549, H1703, and H460) and the patient-derived brain-metastatic lung adenocarcinoma cell line (UW-lung-16) and primary human adenocarcinoma cell line H1792 reveal that all express *GABRA5* (Figure 1B), concordant with RT-PCR analysis (Figure S1C). In Western blot analysis, the expression of the GABRA5 protein band in patient-derived brain-metastatic UW-lung-16 cells appears to be lower compared to the A549 cells. RT-PCR analysis also revealed variable levels of the expression of *GABRA3* mRNA in all NSCLC cell lines (Figure S1D). The immunohistochemistry (IHC) analysis of tissue sections from human lung adenocarcinoma primary tumors and matching brain metastases from the cerebral cortex revealed the membrane and cytoplasmic expression of GABRA5 in large pleomorphic tumor cells along the lining of alveolar walls (Figure 1C left panel) and in larger tumor cells in the cerebrum with well-defined borders and surrounding gliotic tissue (Figure 1C right panel). Additionally, diffuse membrane and the cytoplasmic expression of GABRA3 protein were observed in both primary lung adenocarcinoma and matched brain-metastatic tissue sections (Figure S1E). These findings suggest the potential involvement of *GABRA5* and *GABRA3* in NSCLC progression and metastasis.

### 3.2. Activation of GABA(A) Receptors Is Depolarizing and Triggers Cell Death 

Although enhanced the gene expression and increased protein abundance of GABA(A) receptor subunits suggest the presence of a functional receptor, it is not confirmative. We employed a patch-clamp electrophysiology of single primary-patient-derived adenocarcinoma cells to demonstrate intrinsic, functional GABA(A) receptors. We observe a current signal in response to GABA treatment (Figure 1D). Treatment with benzodiazepine sensitizes GABA(A) receptors in triggering a functional response leading to a potentiation effect of GABA. We observe an enhanced response to GABA plus the benzodiazepine analog AM-101 (1 and 4 µM, respectively) over GABA (1 µM) alone in H1792 cells as detected by patch-clamp electrophysiology (Figure 1D). In this experiment, we utilized an AM-101 concentration of 4 µM since this concentration demonstrated the best potentiation effect in patch-clamp electrophysiology. This functional analysis reveals that H1792 cells possess (1) intrinsic functional GABA(A) receptors, and (2) receptors that form a canonical assembly with an α-γ interface, given that a benzodiazepine can bind and elicit a positive response. 

The benzodiazepine analog employed in our studies, AM-101, is an experimental drug. We have previously reported anti-tumor effects of this compound in multiple cancer settings including medulloblastoma and melanoma [21,22]. In a previous study, we reported that GABA(A) receptor activation by AM-101 leads to an efflux of chloride anions across the extracellular membrane, which contributes to a depolarization of the mitochondrial transmembrane [21]. Similarly, we tested if AM-101 creates a shift in the electric charge distribution of a lung adenocarcinoma cell type. To do so, we employed the cationic fluorescent dye tetramethyl rhodamine ethyl ester (TMRE) and monitored its binding by Fluorescence-Activated Cell Sorting (FACS) [45]. Firstly, compared to the DMSO-treated (control) cells, AM-101 causes a shift in the electric charge distribution (depolarization) of H1792 cell mitochondria. Secondly, this effect occurs rapidly, within 15 min of AM-101 treatment (Figure 1E). Significantly, AM-101-induced depolarization is to an extent equivalent to that induced by treating H1792 cells with the potent mitochondrial oxidative phosphorylation uncoupler FCCP (2-[2-[4-(trifluoromethoxy)phenylhydrazinylidene]-propanedinitrile). AM-101 induces a rapid and significant depolarization of lung adenocarcinoma cells.

Membrane depolarization triggers cell death via the activation of the intrinsic (mitochondrial) apoptotic pathway [46]. Indeed, we have reported this phenomenon in cell lines of the pediatric brain cancer medulloblastoma and melanoma [21,22]. We tested if AM-101 similarly impaired the viability of lung adenocarcinoma cells. AM-101 impairs the viability of not only lung adenocarcinoma cells, including KRAS and TP53 mutated, but of patient-derived cells of other NSCLC subtypes, including squamous cell and large cell, with IC_50_ values in the range of 2–4 μM (Figure 1F; Figure S2A). Cell proliferation impairment occurs after a 72 h incubation with AM-101, which correlates with key events in autophagy, as we will be detailed below. We also determined the IC50 of AM-101 for the patient-derived lung adenocarcinoma brain-metastatic cells UW-lung-16 [32] and report a value like that observed for adenocarcinoma primary cells (Figure 1F and Figure S2A,B). Importantly, our experiments indicate that a single 7-ethinyl in the 1,4-benzodiazepine ring system of AM-101 bestows this cytotoxic response (Figure 1D; Figure S2C), as diazepam (valium) is not cytotoxic to lung adenocarcinoma cells, even at exceedingly higher concentrations (Figure S2C,D). To assess whether AM-101 affects the growth of normal lung cells, we conducted a time-course study using BEAS-2B cells, which are immortalized normal human lung bronchial epithelial cells. We treated BEAS-2B cells at the IC_50_ concentration of AM-101 for H1792 lung cancer cells. Brightfield imaging over a period of 96 h revealed no cytostatic effect of AM-101 on the BEAS-2B culture (Figure S2E).

### 3.3. GABA(A) Receptor Activation Potentiates Radiation

**Figure 2:**
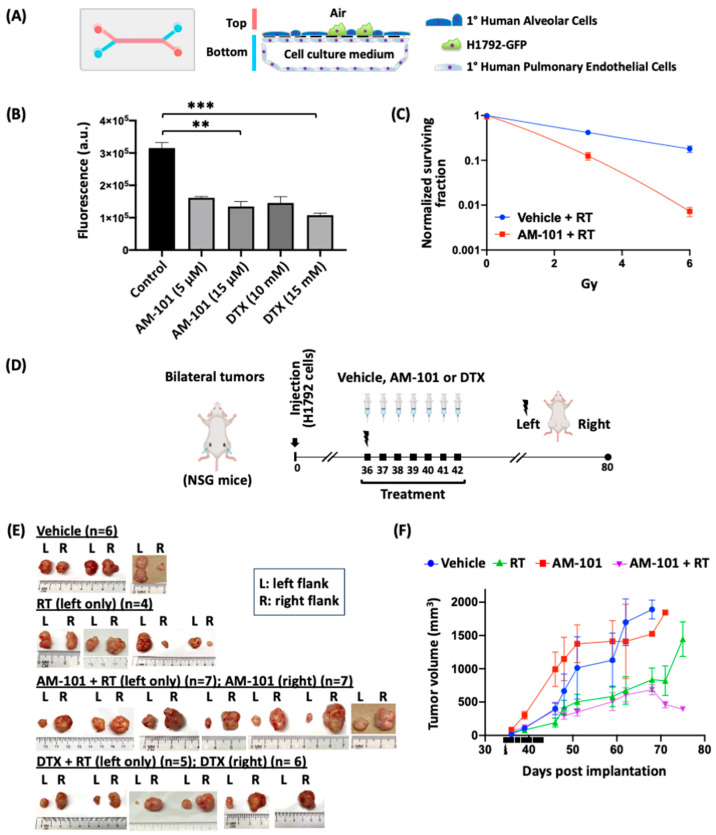
GABA(A) receptor activation potentiates radiation. (**A**) Illustration of a human-relevant ex vivo ‘chip’ employed to test AM-101 and docetaxel (DTX) efficacy. Lung adenocarcinoma cancer cells (H1792-GFP, green) can be co-cultured with primary human alveolar and pulmonary endothelial cells and exposed to air (air–liquid interface) on-chip. Cancer cells form clusters that grow and spread through the epithelial compartment of the chip over time (Figure S3). (**B**) Testing of AM-101 and DTX ex vivo or ‘on-chip’ reveals that AM-101 is as cytotoxic as DTX but at a significantly lower concentration. The chip is a 3-D ex vivo model, and, to achieve the cytotoxicity that AM-101 generates in 5 μM concentration, DTX is required in 10 mM concentrations. To determine *p*-values between two groups, one-way ANOVA with Tukey’s multiple comparisons test was performed. ** *p* < 0.001 and *** *p* < 0.0001. Images acquired from chips were subjected to background signal removal and analysis using Fiji (Image J). To generate bar graphs, acquired images were evaluated through background subtraction and signal thresholding. Subsequently, particle analysis was performed using a Fiji plugin to estimate the number of GFP+ cells per field of view under each testing condition. (**C**) A clonogenic assay was employed to examine the radio-sensitizing effect of AM-101 in H1792 cells. The survival curves showing surviving fraction of H1792 cells following radiation exposure at two separate doses with and without AM-101. Cell cultures were treated with either AM-101 (2.5 μM) combined with two separate doses of radiation (3 Gy and 6 Gy) versus DMSO (vehicle) and two separate doses of radiation (3 Gy and 6 Gy). H1792 cells in culture were treated with AM-101 (2.5 μM) or DMSO (vehicle) 1 h before radiation and maintained in the medium after irradiation. According to the experimental design the media containing AM-101 or DMSO in all groups was replaced with fresh media 72 h after treatment. Colony-forming efficiency was determined 14 days later, and survival curves were generated. The vehicle in this experiment is DMSO, since DMSO is used as the solvent to solubilize AM-101. (**D**) Schematic of the efficacy experiment in H1792 subcutaneous heterotopic bilateral xenograft tumors generated in NSG mice. Mice in vehicle or drug treatment groups received i.p., vehicle, AM-101 (2.5 mg/kg), or DTX (8 mg/kg), on day 36 post-implantation and then six injections once per day. Mice in radiation (RT) or combo groups received a single fraction of radiation (5 Gy) to left flank only at 2 h before vehicle or drug on the first day of treatment. (**E**) At experimental endpoint, tumors from left (L) and right (R) flanks of each mouse were resected. H1792 subcutaneous xenograft tumor growth in NSG mice from different treatment groups: vehicle, radiation (RT), AM-101 ± RT, DTX ± RT. Number of mice per treatment group: *n* = 6 for vehicle, *n* = 4 for RT; *n* = 7 for AM-101 and *n* = 7 for AM-101 + RT, *n* = 5 for DTX + RT and *n* = 6 for DTX. (**F**) Tumor volume of left and right flank tumors was measured over time using Vernier calipers. The tumor growth delay curves show the tumor volumes of mice treated with a vehicle, radiation (RT), AM-101, and AM-101 plus RT. Each point on the curve represents the mean tumor volume after treatment, with error bars indicating the standard error (SE). Statistical significance is indicated by *p* < 0.001.

Since AM-101 is cytotoxic to lung cancer cells, albeit in the low micromolar range (2.5–3.5 μM), we further investigated its potency and whether it was as efficacious as the anti-microtubular agent docetaxel (Taxotere), a chemotherapeutic commonly employed clinically as a radiation sensitizer for advanced-stage NSCLC. Firstly, we employed an ex vivo micro-scale ‘chip’ composed of human-derived alveolar lung cells, lung endothelial cells, and GFP-tagged H1792 cells to provide an assay with a readout of efficacy that, to a degree, recapitulates the human lung tumor microenvironment (Figure 2A). ‘Chips’ co-cultured with H1792-GFP cells exhibit staining for phalloidin (epithelial cell marker) and PECAM1 (endothelial cell marker) (Figure S3A,B). H1792-GFP cells grow and spread through the epithelial compartment of the ‘chip’ over time (Figure S3C,D). H1792-GFP cells are killed by docetaxel in a concentration-dependent manner, as seen by a reduction in the number of GFP positive cells in the chip (Figure S3E,F). However, this analysis revealed that AM-101 is equally as efficacious as docetaxel in terms of its effect on H1792 cell killing but effective at a significantly lower concentration than docetaxel (Figure 2B). While a concentration of 2.5 µM of AM-101 was found to be cytotoxic for H1792-GFP cells, the data presented in this study represent the optimal cytotoxicity achieved with AM-101 in an ex vivo chip model in which lung cancer cells are co-cultured with human bronchial endothelial and bronchial epithelial cells. The effective concentration of AM-101 for the ex vivo chip model is higher than the IC_50_ determined by the MTS assay. The MTS assay employs a 2D culture with H1792 cells only and does not represent the human tumor microenvironment (TME). Lower GFP intensities compared to the control indicate cell loss due to the cytotoxic effects of the treatment agent (AM-101 or docetaxel). In contrast, the 3D cell-co-culture system better recapitulates the tumor microenvironment, hence requiring a higher concentration of AM-101 than the observed IC_50_ values obtained by the MTS assay. Although 2.5 µM of AM-101 induced some cytotoxicity in the chip model, the 5 µM concentration elicited an optimal cell killing in this system. 

We then explored the ability of AM-101 to function as a radiation sensitizer of lung adenocarcinoma cells. AM-101 was found to rapidly permeate the blood–brain barrier in 30 min, and no side effects including neuropathic pain have been reported when this agent was used in rodents and monkeys [47]. Using a clonogenic assay with lung adenocarcinoma H1792 cells, we observe a significant effect of AM-101 alone on impairing clonogenicity (Figure S4A,C). However, AM-101 when combined with radiation has a greater inhibitory effect on H1792 cell clonogenicity than either radiation or AM-101 alone (Figure 2C and Figure S4A,C). As the radiation dose increases, there is a marked reduction in surviving fraction of cells in the radio-sensitized group compared to the control (vehicle) group (Figure 2C). At higher radiation doses the difference between the two curves becomes more pronounced, demonstrating the enhanced sensitivity of the H1792 cells to radiation when pre-treated with AM-101.

This greater potency of AM-101 plus radiation provided the impetus to pursue mouse efficacy studies of AM-101 alone and in combination with radiation. We generated heterotopic xenograft bilateral (left and right flank) tumors in NOD scid gamma (NSG) mice using H1792 cells (Figure 2D), as this adenocarcinoma cell line produces tumors that are poorly responsive to radiation [48]. Our treatment protocol involved administering a single i.p. daily dose of AM-101 (2.5 mg/kg) or docetaxel (8 mg/kg) for seven consecutive days, once the tumor was palpable. For those mice in radiation-receiving treatment groups, since we did not treat the tumor with multiple fractionated radiation doses, a single dose of radiation (5 Gy) was administered exclusively to the left-flank tumors of mice only on the first day of AM-101 or docetaxel i.p. injection. We analyzed tumor weight at the experimental endpoint and tumor growth delay over time by measuring the tumor volume. The gross visual inspection of left (irradiated) vs. right (non-irradiated) resected tumors highlights the following: (1) radiation alone does not exhibit a clear, consistent effect on final tumor size; (2) AM-101 or docetaxel alone (right flanks) does not contribute to significant tumor control relative to vehicle; (3) the combined treatment of AM-101 or docetaxel with radiation (left flanks) lowered the mean weight of the xenograft tumor in mice at the experimental endpoint and resulted in consistently smaller tumor sizes (Figure 2E; Figure S5A). In addition, there is no significant difference between median days to the endpoint of control-vehicle group mice versus those receiving radiation. However, there is a significant difference for mice in the combined (AM-101 plus radiation group) (Figure S5B). Docetaxel also results in a statistically similar effect when combined with radiation (Figure S5A). Thus, both therapeutic agents appear to radiation sensitize equally as well. Indeed, this is the basis for why docetaxel has been employed clinically as a radiation sensitizer. The analysis of tumor growth delay over time reveals that AM-101 plus radiation delayed mice tumor growth compared with vehicle control, drug alone, or radiation alone (Figure 2F). This occurs even though treatment involves only a single radiation dose with seven days of AM-101 administration early in the appearance of tumor formation. The slope of the curves of the radiation versus combined treatment groups appear similar up to ~ 68 days, at which point they diverge, suggesting that combined treatment has a protracted impact on tumor growth. This effect is manifested as an increase in median days to the endpoint for those mice receiving the combination treatment, AM-101 plus radiation (Figure S5B). 

### 3.4. Increased Survival of Mice Bearing a Lung Brain-Metastatic Tumor

The analysis of AM-101 and docetaxel in the context of heterotopic tumors revealed that each works equally as well in potentiating radiation. As highlighted in the introduction, there is a significant demand for radiation-sensitizing agents that are brain-penetrant and non-toxic to improve the standard of care for lung-cancer-associated brain metastasis treatment [6]. Docetaxel is not brain-penetrant and, therefore, not employed to treat lung-cancer-associated brain metastasis. Docetaxel is also associated with significant co-morbidities, including neuropathic pain. 

**Figure 3:**
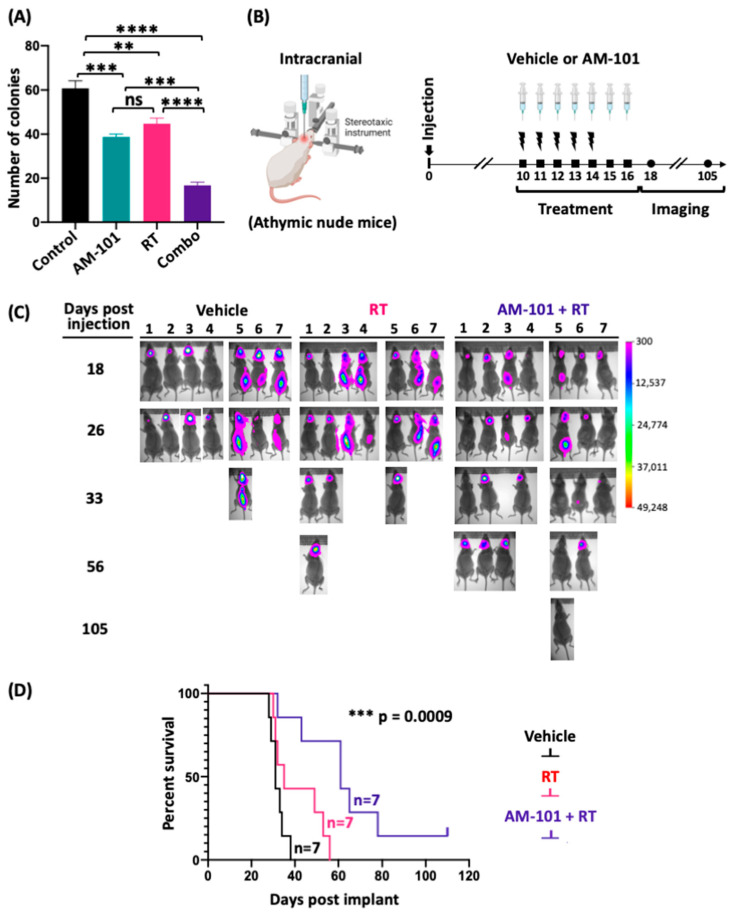
GABA(A) receptor activation increases survival of mice bearing lung brain-metastatic tumors. (**A**) Bar graphs representing the number of colonies generated from clonogenic assay to determine the radiosensitizing effect of AM-101 when combined with radiation treatment (RT) versus AM-101 (2.5 μM) or RT (3 Gy) alone in patient-derived brain-metastatic UW-lung-16 cells. The combination of AM-101 plus RT (combo group) imparts the most significant impact on colony formation, a three-fold suppression of colony numbers than control and two-fold suppression of colony numbers than AM-101 and RT applied alone. Control is DMSO treated, as DMSO is the diluent of AM-101. Data are represented as mean ± S.E. One-way ANOVA with Tukey’s multiple comparisons test was performed to determine the *p*-values between two treatment groups. The one-way ANOVA *p* < 0.0001. Based on Tukey’s multiple comparisons test, Control vs. AM-101, *** *p* = 0.0001; Control vs. RT ** *p* = 0.0019; Control vs. Combo **** *p* < 0.0001; AM-101 vs. RT, ns (not significant), *p* = 0.308; AM-101 vs. Combo *** *p* = 0.0001; RT vs. Combo **** *p* < 0.0001. (**B**) Schematic of efficacy experiment in intracranial xenograft tumors generated in athymic nude mice. Mice received a stereotaxic intracranial injection of cells (UW-lung-16) from a brain lesion of a patient with lung cancer. Mice (*n* = 21) were separated into three treatment groups (*n* = 7 per group). Ten days post-injection, mice received (1) vehicle, an i.p. injection of formulation; (2) radiation (RT), 2.5 Gy dose/day to the whole mouse brain for 5 consecutive days; (3) AM-101 plus RT, i.p. injection of formulated AM-101 (5 mg/kg) and RT (2.5 Gy dose/day/mouse for 5 days) to the whole mouse brain using a XenX irradiator (Xstahl Ltd.) and the supplied mouse gantry. The day of intracranial injection of tumor cells was assigned as day zero. Tumors were followed by bioluminescent imaging (BLI) over time. (**C**) BLI study of mice with brain metastatic lung tumors and treated with vehicle, radiation (RT), or RT plus AM-101. Mice were imaged at indicated time points post-intracranial injection of tumor cells. (**D**) Kaplan–Meier survival curve with *p*-value (log rank test) calculated for statistical significance. Kaplan–Meier curves were used to estimate the survival of mice in each group in mouse intracranial xenograft experiments. Statistical significance was determined by using the log-rank test.

As discussed, and shown above, GABA(A) receptors are present in both patient adenocarcinoma primary and brain-metastatic lesions. We therefore explored if AM-101 is effective in potentiating radiation in the brain metastasis setting. We first examined the effect of AM-101 on clonogenicity of a patient-derived brain-metastatic line, UW-lung-16. AM-101 alone impairs the viability of these cells and significantly inhibits their clonogenicity (Figure 3A; Figure S4B). RT alone has some effect on colony formation, but AM-101 has a greater effect on suppressing the clonogenic ability of UW-lung-16 cells. When combined with radiation, the effect of suppressing the growth of colonies is significantly (about 2-fold) better than AM-101 or radiation treatment (RT) alone. The combination significantly reduced the number of individual colonies in the clonogenic assay. Second, we generated UW-lung-16 luciferase-tagged cells from the parental cell line and employed this line for in vivo experiments. We generated intracranial xenograft tumors by stereotaxic injection in athymic nude mice and monitored tumor growth by bioluminescence imaging (BLI), symptoms of mice, and overall survival (Figure 3B). This experiment entailed a treatment consisting of seven-day consecutive i.p. injections of AM-101 (5 mg/kg/day) and a five-day consecutive whole-brain radiation exposure (2.5 Gy/day), overlapping with the first five days of AM-101 administration (Figure 3B). In the control mice treated with vehicle (a co-solvent formulation), tumors develop within 18 days post-implantation, as visualized by bioluminescence imaging (BLI) (Figure 3C). The fractionated whole-brain radiation dose of 2.5 Gy/day per mouse was selected based on a previously published radiosensitizer study performed with the same UW-lung-16 intracranial metastatic mouse model [5]. Tumors caused severe neurologic symptoms in mice by day 26, which manifested as impaired mobility and a hunched physical appearance (see Supplementary Videos, Video S1). Kaplan–Meir survival analysis shows that whole-brain radiation provides no survival benefit, which is corroborated by luciferase imaging (Figure 3C,D) and in video recordings of symptoms in mice (Supplementary Videos, Video S1). In contrast, mice treated with AM-101 plus radiation show a significant survival benefit, and 70% of mice survived beyond radiation-alone-treated mice (Figure 3C,D). The median days to the endpoint of mice treated with AM-101 plus radiation was markedly higher than mice treated with vehicle and radiation only, 61 days versus 35 days, respectively (Figure S5B). One mouse did not exhibit any evidence of the recurrence of an intracranial tumor by luciferase imaging (Figure 3C, at 105 days post-injection), nor did it at the end of the study when the brain was resected to identify macroscopic evidence of tumor. The mean flux intensity from the brains of mice, as analyzed from bioluminescent images, was significantly lower in the group treated with the combination of AM-101 and radiation compared to the group treated with radiation alone on days 26, 33, and 56 (Figure S5C). There was no significant change in the body weight of AM-101-plus radiation-treated mice during the entirety of the experiment, indicating no added toxicity (Figure S5D). We note that about half of all mice in each treatment group had cancerous cells in the spinal region (Figure 3C). Interestingly, mice receiving radiation localized to the brain plus AM-101 exhibited a loss of tumor cells in the brain as well as spine. Further studies are required to understand the effect of AM-101 with radiation on possible spinal or leptomeningeal metastasis. Since our heterotopic xenograft mouse experiments demonstrated that AM-101 alone does not provide significant tumor control in immunocompromised NSG mice, we did not include a group treated with AM-101 alone in our mouse intracranial metastatic xenograft experiments. 

### 3.5. GABA(A) Receptor Activation Enhances Autophagic Puncta and GABARAP and Nix Multimerization

Having analyzed the effectiveness of AM-101 to potentiate radiation in two mouse tumor models, we examined the contribution of molecular events to the observed effect. In considering how the AM-101 activation of GABA(A) receptors may mediate adenocarcinoma cell death and tumor control, several observations by us and others suggested that autophagy may be a contributing factor. First, we reported above that AM-101 activation depolarizes the mitochondrial transmembrane. It has been reported that the depolarization of mitochondria serves to trigger autophagy [38]. Second, a central protein to autophagy induction is GABARAP, which, prior to the elucidation of its role in autophagy, was shown to interact with GABA(A) receptors [26]. GABARAP is also a Nix-interacting factor, which has a significant role in autophagosome formation [29]. Third, our immunoblotting experiments showed that AM-101 treatment in vitro does not enhance cleavage of pro-caspase 3 in lung adenocarcinoma cells (Figure S4D) even at concentrations higher than its IC_50_. Cleaved or activated pro-caspase 3 contributes to the inhibition of autophagy by catalyzing the proteolysis of key autophagy-associated proteins, including Beclin-1, ATG5, and p62 [49,50].

**Figure 4:**
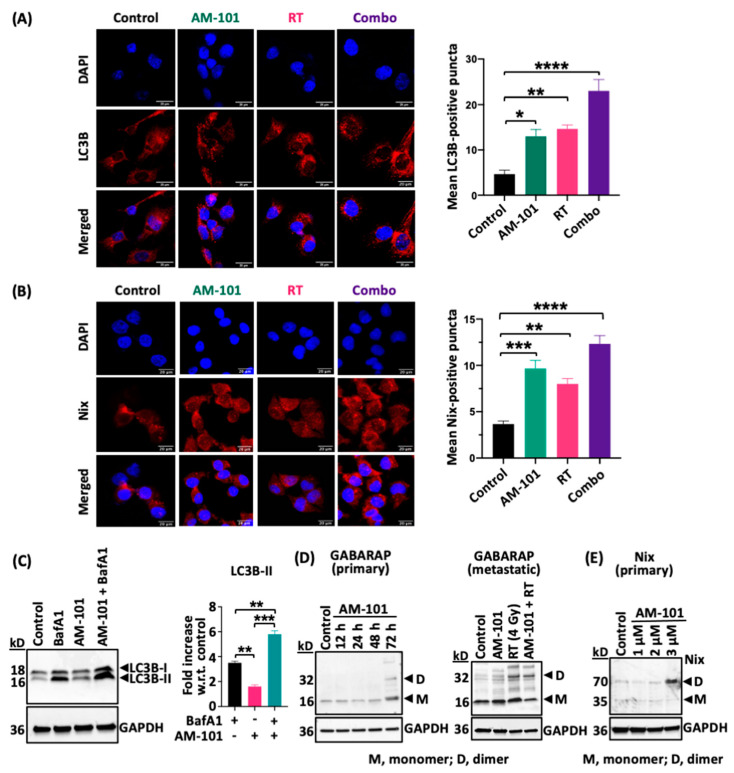
GABA(A) receptor activation enhances autophagic puncta and the flux and triggers multimerization of GABARAP and Nix. (**A**) Shown are confocal immunofluorescence microscopic images of H1792 cells under various treatments: DMSO or control; AM-101 (3 µM); radiation (RT); and AM-101 plus RT (combo) (scale bar, 20 µm). Radiation dose was 3 Gy. Cells were stained for DNA with DAPI (blue fluorescent) and LC3B (left) using LC3B antibody. LC3B puncta were quantified per 3 cells for each experimental group and plotted, as shown in the bar graph, where * *p* = 0.0108 (control vs. AM-101); ** *p* = 0.0037 (control vs. RT); **** *p* < 0.0001 (control vs. combo) (right), which reveals a similar effect between AM-101 versus RT, but combining these two has a statistically pronounced impact on puncta formation. (**B**) Confocal immunofluorescence microscopic images of H1792 cells under various treatments that were then stained for DAPI and Nix to identify and quantify Nix puncta (left). Nix puncta were quantified per 3 cells for each experimental group and plotted, as shown in the bar graph, where *** *p* = 0.0008 (control vs. AM-101); ** *p* = 0.006 (control vs. RT); **** *p* < 0.0001 (control vs. combo) (right), which reveals a pronounced effect of AM-101 on puncta formation and an increase in puncta when AM-101 is combined with radiation (RT) (combo treatment group). For statistical calculations, one-way ANOVA was performed and followed up with Dunnett’s multiple comparisons test. (**C**) Immunoblotting (using 4–15% gradient gel PAGE) demonstrates enhanced autophagic flux with LC3B-II as a marker in H1792 cells following co-treatment with AM-101 and bafilomycin A1. A representative immunoblot probed with an LC3B antibody shows the results from cell lysates of control (DMSO-treated) and three treatment groups: BafA1 alone, AM-101 alone, and AM-101 combined with BafA1. H1792 cells were treated with 3 μM AM-101 for 48 h, followed by either 50 nM bafilomycin A1 (AM-101 + BafA1) or DMSO (vehicle) for an additional 4 h. Control cells were treated with DMSO for 48 h and then with either 50 nM bafilomycin A1 or DMSO for 4 h. The right panel shows LC3B-II band intensities quantified using ImageJ, with bar graphs representing the fold increase in LC3B-II for each treatment group relative to the vehicle control (data shown as mean ± SEM, *n* = 2). Co-treatment of AM-101 and bafilomycin A1 significantly increased LC3B-II compared to AM-101 or bafilomycin A1 alone. GAPDH is used as loading control. To measure the statistical significance, ordinary one-way ANOVA (one-way ANOVA *p* < 0.0006) with Tukey’s multiple comparison test was performed to compare the means of each group. ** *p* = 0.0051 (BafA1 vs. AM-101); ** *p* = 0.0029 (BafA1 vs. AM-101 + BafA1); and *** *p* = 0.0005 (AM-101 vs. AM-101 + BafA1). (**D**) Modified immunoblotting of SDS gels (4–15% gradient gel) showing the effect of AM-101 on monomeric GABARAP expression and its oligomeric state in H1792 cells. (**E**) Modified immunoblotting of SDS gels (4–15% gradient gel) showing the effect of AM-101 on monomeric Nix protein expression and its oligomeric state in H1792 cells. AM-101 (3.0 µM) triggers a multimerization of GABARAP and an apparent increase in abundance at 72 h (left). Nix abundance is also enhanced as well as formation of dimer by AM-101 (3 µM) in a concentration-dependent manner in H1792 cells. GAPDH is used as a loading control for both experiments in (**D**,**E**). D: dimer; M: monomer. Original western blots are presented in Figure S9.

Key proteins to the assembly of autophagy granules or puncta, a hallmark of autophagy induction, are the ATG8 sub-family-associated protein LC3B and Nix. Importantly, Nix mediates an association between the mitochondria and GABARAP, which is associated with the GABA(A) receptor at the extracellular membrane. Subcellular distribution of autophagosomal proteins by immunofluorescence (IF) (e.g., LC3 puncta formation) is a well-established assay to monitor autophagy since LC3B is a general marker for autophagic membranes during the maturation of autophagosome [51]. It is known that lipidated LC3B (LC3B conjugated with phosphatidylethanolamine), which is present at the autophagosomal membrane, is observed as puncta within the cell cytoplasm, whereas poorly lipidated LC3B shows a more diffuse staining [50]. We therefore employed confocal IF to observe the assembly of puncta in lung adenocarcinoma H1792 cells treated with AM-101 and radiation alone as well as the two combined. There is a background level of both LC3B and Nix-positive puncta in H1792 cells in the control (DMSO treated) group (Figure 4A,B). However, quantification of puncta reveals that AM-101 alone creates an environment for increased puncta positive for both markers (Figure 4A,B; see bar graphs). Similarly, radiation of cells contributes to increased puncta formation. However, the most significant increase in puncta is observed when AM-101 and radiation are combined.

Next, we investigated whether AM-101 treatment increases the autophagic flux in H1792 lung adenocarcinoma cells. Using bafilomycin A1, a lysosomal proton pump inhibitor that disrupts the autophagic flux, we measured autophagic flux by assessing LC3B lipidation (conversion of LC3B-I to LC3B-II). Autophagic flux is measured by the accumulation of LC3B-II, the lipidated form of LC3B that associates with autophagosomal membranes. Since the enhancement of LC3B-II flux typically refers to an increase in the autophagic process, we analyzed Western blot data using the densitometry of LC3B-II protein band, the lower band in the Western blot of LC3B. Our results show that treating H1792 cells with AM-101 (2.5 µM for 72 h) in combination with bafilomycin A1 (50 nM for 4 h) elicits an approximately 6-fold increase in LC3B-II accumulation compared to control, DMSO alone (Figure 4C). This increase is about twice that of cells treated with bafilomycin A1 alone (Figure 4C). These findings indicate that AM-101 treatment enhances autophagic flux in support of AM-101 functioning as an autophagy inducer (Figure 4C).

After observing an increased assembly of LC3B and Nix-positive puncta, along with enhanced autophagic flux, we focused on the protein GABARAP since Nix binds specifically to GABARAP. In a time-course experiment, H1792 cells treated with AM-101 show a pronounced accumulation of both GABARAP and Nix at 72 h post-treatment. GABARAP forms dimers and higher-order polymers in neural cells, such as PC12 cells [52]. Previously, these dimers and polymers have been detected in immunocomplexes from PC12 cells when analyzed on SDS-PAGE gels [42]. In our experiment, we utilized a modified Western blot protocol, which involves omitting beta-mercaptoethanol from the sample buffer and refraining from heating the samples before loading them onto a polyacrylamide gel. Importantly, we observed that GABARAP and Nix also dimerize at 72 h (Figure 4D,E) post treatment with AM-101. This is the time point when lung adenocarcinoma cell viability is impaired significantly by AM-101. Furthermore, AM-101-induced Nix dimerization is dose-dependent (Figure 4E). Nix (BNIP3L) forms stable homodimers, which are more efficient at recruiting autophagosomes compared to their monomeric form [53]. It has been established that Nix (BNIP3L) dimerization enhances its activity as a mitophagy receptor, thereby promoting the autophagic clearance of mitochondria [53]. The Nix dimerization induced by AM-101 treatment in H1792 cells is a crucial indicator of initiation of autophagosome recruitment. Previous studies have detected Nix dimers, along with monomers, using SDS-PAGE followed by immunoblotting with a Nix antibody [54]. The interaction of GABARAP dimers with Nix dimers is a later step in the autophagosome formation stage, which may explain why enhanced dimers were not detected in earlier time points. We also observed that radiation alone, as well as radiation plus AM-101, triggers GABARAP dimerization in vitro in brain-metastatic cell line UW-lung-16, corroborating the observation in primary cells (Figure 4D). However, we did not observe an accumulation of multimers of these proteins in tumor samples. This may stem from the fact that multimers are non-covalent complexes and most likely disrupted during tissue processing. Also, the tumors were resected at the end of the experiment when tumors have recurred.

### 3.6. Enhanced Levels of Autophagy Biomarkers in Cells and Tumors

Having established that GABA(A) receptor activation leads to (1) the enhanced formation of LC3B and Nix-positive puncta and (2) GABARAP and Nix time-dependent multimerization, we turned to examine if GABA(A) receptor activation elicited an effect on the abundance and activity of other proteins key to various aspects of autophagosome assembly. Specifically, we focused on proteins ATG7, Beclin-1, and p62, the autophagy substrate.

**Figure 5:**
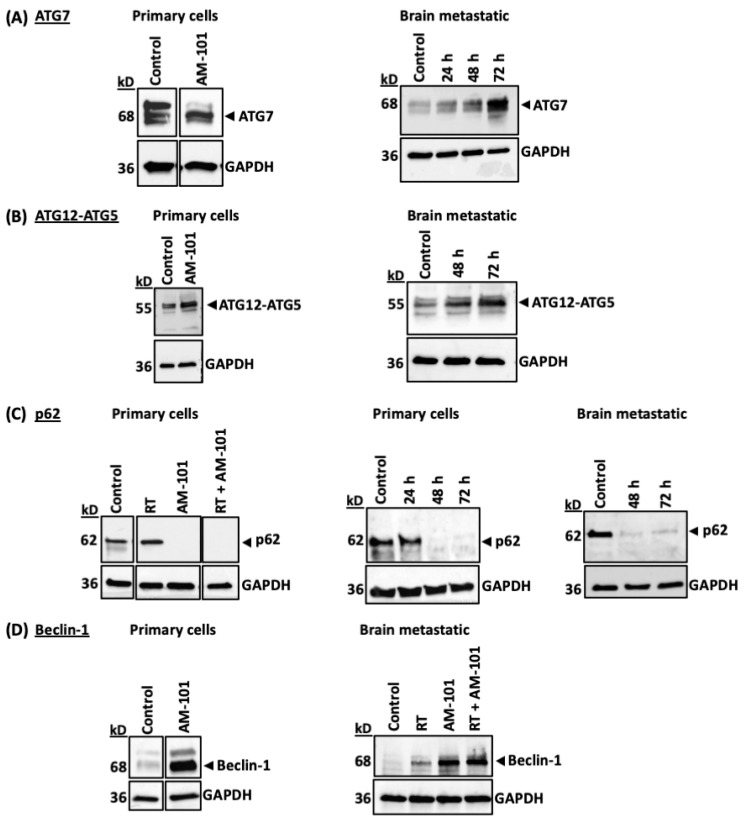
Change in abundance or utilization of autophagy biomarkers in response to GABA(A) activation. (**A**) ATG7 immunoblot (4–15% gradient polyacrylamide gel) of patient-derived lung adenocarcinoma primary (H1792) and brain-metastatic (UW-lung-16) cells following treatment with AM-101. Left, immunoblot showing increased expression of one isoform of ATG7 protein in primary H1792 cells of following treatment with 3.0 µM of AM-101 for 48 h compared to the control. Control: DMSO treated. Right, immunoblot showing change in ATG7 protein levels over time in lung cancer brain-metastatic UW-lung-16 cells treated with 3.0 µM AM-101 compared to the control. Significant increase in ATG7 protein is observed at 72 h. Control: DMSO treated. (**B**) Immunoblots showing the effect of in vitro AM-101 treatment on protein levels of ATG-12-ATG5 conjugate in primary H1792 cells (**left** panel) and patient-derived lung cancer brain metastatic UW-lung-16 (**right** panel) cells. In case of H1792 cells (**left** panel) sample was collected at 72 h after AM-101 treatment and in case of UW-lung-16 cells (**right** panel), samples were collected from both 48 h and 72 time points post treatment. (**C**) p62 immunoblots of SDS gels of lung adenocarcinoma primary (H1792) and brain-metastatic (UW-lung-16) cells following treatment with AM-101. Left, changes in expression of p62 protein as assessed by immunoblotting of lysates from H1792 cells treated with AM-101, radiation (3 Gy), and a combination of radiation (RT) plus AM-101 only. Control, DMSO. Middle, evaluation of the time-dependent utilization of p62 after AM-101 (3.0 µM) treatment in primary lung cancer cell (H1792). Right, patient-derived brain-metastatic lung adenocarcinoma cell line UW-lung-16 (right). Control, DMSO. GAPDH is used as a loading control. (**D**) Left, immunoblot showing Beclin-1 protein levels in control and AM-101 treated H1792 cells (treated for 72 h). Control, DMSO. Right, immunoblot shows Beclin-1 protein levels in patient-derived lung brain-metastatic UW-lung-16 cells treated with AM-101, radiation (3 Gy), and radiation (3 Gy) along with AM-101. Original western blots are presented in Figure S10.

ATG7 is a ubiquitin-activating E1-like enzyme that drives autophagosome formation [55,56,57]. AM-101 in vitro enhances ATG7 protein levels in both patient-derived adenocarcinoma primary (H1792) and brain-metastatic (UW-lung-16) cells (Figure 5A). During autophagy, ATG7 facilitates the conjugation of ubiquitin-like protein ATG12 to a lysine of ATG5, generating an ATG12-ATG5 conjugate [58]. This ATG12-ATG5 conjugate is essential for subsequent stages of autophagosome formation. We therefore examined if AM-101 treatment of primary H1792 cell and lung cancer brain metastatic UW-lung-16 cells enhances the formation of an ATG12-ATG5 conjugate. Western blotting using an antibody against ATG12 shows a 55 kD product, which reflects the size of the ATG12-ATG5 conjugate. Detection of this ATG12-ATG5 conjugate protein is expected with this ATG12 antibody as per the antibody datasheet (Cell Signaling Technology, catalog # 4180). AM-101 in vitro treatment enhances the ATG12-ATG5 conjugate in both primary (H1792) and brain-metastatic (UW-lung-16) NSCLC cells (Figure 5B). 

Interestingly, of three splice variants of ATG7, the observed change in the primary cells is of a variant functionally associated with autophagy induction (see Figure 5A, primary cells). Change in ATG7 levels is not only observed in H1792 cells; the immunoblotting of heterotopic H1792 tumor tissue lysate shows a nominal degree of enhanced ATG7 staining in mice treated with AM-101 (Figure S6A). 

Sequestosome-1(SQSTM1)/p62 or p62 is a ubiquitin-binding multifunctional protein that acts as a selective autophagy substrate [59]. AM-101 alone induces a depletion of autophagy substrate protein p62, which is indicative of its utilization (Figure 5C). In contrast, there is no change in the level of p62 in H1792 cells when exposed to radiation (3 Gy) alone. However, radiation plus AM-101 depletes p62 (Figure 5C). In a time-course experiment, we found that p62 is depleted within 48 h after AM-101 treatment in primary (H1792) and brain-metastatic lung adenocarcinoma lines (UW-lung-16) (Figure 5C). p62 serves as a specific substrate for autophagy, and the initiation of autophagosome formation is consistently primed by the recruitment of p62 [60]. This reinforces our observation of an early utilization of p62, which begins at 48 h post-treatment and persists at 72 h. We also observed a reduction in the levels of the autophagy substrate p62 protein in tumor tissue lysates from the radiation-only group and the group treated with radiation plus AM-101 compared to tumors from the vehicle-treated group (Figure S6B). 

Finally, Beclin-1 is an autophagy initiator and regulator [61]. In culture, AM-101 enhances Beclin-1 protein expression in primary (H1792) and brain-metastatic (UW-lung-16) cells (Figure 5D). In contrast, relative to AM-101, we observe a marginal increase in Beclin-1 following radiation exposure (Figure 5D). Beclin-1 protein abundance is also enhanced in H1792 heterotopic xenograft tumors from mice treated with radiation, AM-101 alone, and a combination of both (Figure S6C). Importantly, the enhanced abundance of Beclin-1 is AM-101 dependent, as the chemically similar benzodiazepine diazepam (valium) does not enhance Beclin-1 expression (Figure S6D). As noted above (Figure S2C,D), diazepam is not cytotoxic to this cancer line. 

### 3.7. AM-101 Cytotoxicity Is Inhibited by Abrogating the GABARAP–Nix Axis

We observed that primary and brain-metastatic lung cancer cells have enhanced the abundance or utilization of key autophagy proteins when treated with GABA(A) receptor activator, AM-101. Our attention then turned to whether the autophagic response observed contributed to the cytotoxicity of AM-101 and the importance of GABA(A) receptor activation in triggering autophagic events. We tested autophagy inhibitor bafilomycin A1 at a low concentration (10 nm) to see if it would counteract the effect of AM-101. Bafilomycin A1 and AM-101 when combined are no more cytotoxic than each alone (Figure S7A). This suggests that they are involved in a molecular ‘tug-of-war’ on opposite sides in their molecular mechanisms. We also found that bafilomycin A1, when combined with AM-101, counteracts the molecular effects reported above of AM-101, including a reduction in ATG7 abundance as well as the utilization of p62 (Figure S7B,C). These observations indicate that AM-101’s cytotoxic effect on lung adenocarcinoma cells is via the induction of autophagy. However, the high cytotoxic effect of bafilomycin A1 itself on lung adenocarcinoma cells, even at 10 nM (Figure S7A), partially hinders our ability to make a clear conclusion. 

**Figure 6:**
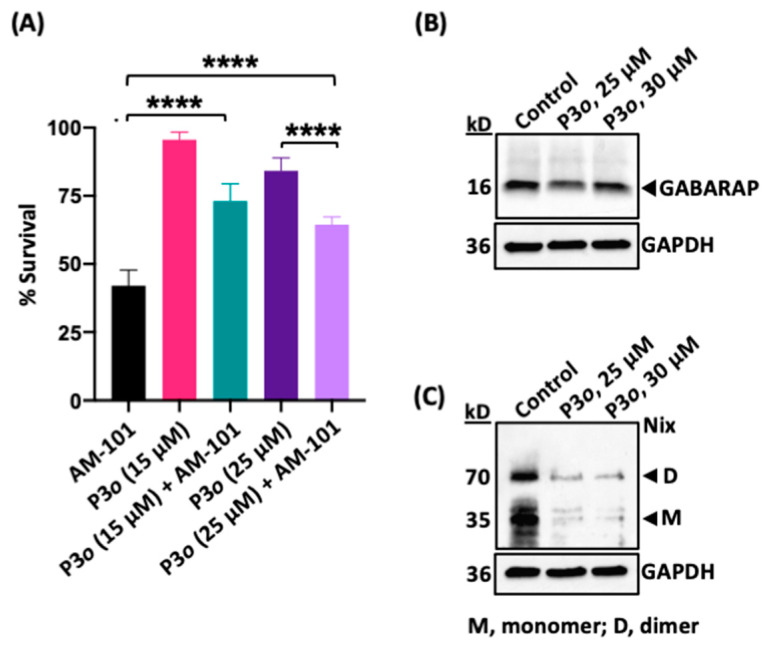
GABARAP–Nix abrogation inhibits AM-101 cytotoxicity. (**A**) Combined treatment of lung adenocarcinoma (H1792) cells with AM-101 plus Pen3-*ortho* (P3*o*), a stapled-peptide that binds to GABARAP and abrogates Nix binding, inhibits the cytotoxicity of AM-101. The inhibitory effect of P3*o* is enhanced with an increased concentration of the inhibitor. **** *p* < 0.0001 [AM-101 vs. P3*o* (15 μM) + AM-101]; **** *p* < 0.0001 [AM-101 vs. P3*o* (25 μM) + AM-101]; **** *p* < 0.0001 [P3*o* (25 μM) vs. P3*o* (25 μM) + AM-101]. One-way ANOVA with Tukey’s multiple comparisons test was performed. (**B**) Treatment of H1792 cells with two different concentrations of P3*o* does not impact GABARAP protein abundance, as observed by immunoblot of SDS gel probed for GABARAP. (**C**) Treatment of H1792 cells with P3*o* reduces both Nix dimer and monomer protein levels, as observed by immunoblot of SDS gel probed for Nix. D: dimer, M: monomer. GAPDH is used as a loading control. Original western blots are presented in Figure S11.

We therefore explored potential ways to inhibit autophagy without eliciting a cytotoxic response. We also desired an approach that had a clear target and mode of action. We employed a small peptide that had been designed to target the protein GABARAP and inhibit autophagy in ovarian cancer cells by blocking Nix binding [9]. This peptide, Pen3-*ortho*, has a couple of advantages: (1) it is highly specific for GABARAP, binding with a low nanomolar affinity; (2) a crystal structure has been determined of Pen3-*ortho* in complex with GABARAP, and its mode of action in blocking autophagy in cancer cells is delineated [9]. Mechanistically, Pen3-*ortho* binds to a site on GABARAP that is disruptive to its interaction with Nix, i.e., functions as a competitive inhibitor. We found that unlike bafilomycin A1, Pen3-*ortho* alone does not exhibit cytotoxic effects on H1792 cells, even at high concentrations (up to 25 μM), as shown in Figure 6A. Previously, a sublethal dose of 25 μM Pen3-*ortho* was tested in an ovarian cancer cell line (OVCAR3) [9]. We further investigated whether Pen3-*ortho*, at two different sublethal concentrations (15 and 25 μM), could inhibit the cytotoxic response of AM-101. We found that the combination of Pen3-*ortho* with AM-101 significantly inhibits the cytotoxicity of AM-101 on lung adenocarcinoma H1792 cells. Our findings demonstrate that the inhibitory effect of Pen3-*ortho* on cytotoxicity is concentration-dependent, with 15 μM of Pen3-*ortho* identified as a sublethal dose on human lung adenocarcinoma H1792 cells. Although the higher dose of 25 μM, previously found to be sublethal in OVCAR3 cells [9], exhibits some cytotoxicity in H1792 cells, it still significantly reverses the cytotoxic effects of AM-101 (Figure 6A). Notably, the lower concentration (15 μM) of Pen3-*ortho* shows a more robust effect in reversing the cytotoxic effects of AM-101 (Figure 6A). As expected, given its nanomolar affinity for GABARAP protein, Pen3-*ortho* does not alter GABARAP protein levels, even at concentrations well above its reported inhibitory concentration (Figure 6B). The Western blotting of H1792 cells treated with Pen3-*ortho* shows a significant reduction in Nix protein, both in monomer and dimer states in treated cells, clearly indicating that Pen3-*ortho* treatment inhibits autophagy (Figure 6C). This supports previously published work that GABARAP interacts with and stabilizes Nix [29] and provides evidence that co-treatment with a GABARAP-specific autophagy inhibitor leads to the reversal of the autophagy-mediated cytotoxic effect of AM1-101. Importantly, these experiments support GABARAP as a mediator between GABA(A) receptor function and autophagosome assembly (see Figure S7D for an illustration of this series of experiments and model based on the effect). 

### 3.8. Contribution of γ-H2AX to Radiation Sensitization

A protein that is indicative of radiation effectiveness, as well as contributing to autophagy, is the phosphorylated form of histone H2AX or γ-H2AX [62,63]. Recently, it was reported that in embryonic stem cells, the activation of GABA(A) receptors using the agonist muscimol increases γ-H2AX in response to double-strand breaks [20,64]. Similarly, we found that AM-101 enhances γ-H2AX in lung adenocarcinoma cells (Figure S4E). Furthermore, γ-H2AX enhancement is augmented when AM-101 is combined with radiation in vitro (Figure S4E). AM-101 may therefore potentiate radiation via γ-H2AX. However, the AM-101 enhancement of γ-H2AX is not observed in the lung cancer brain-metastatic cell line UW-lung-16, although radiation alone does enhance γ-H2AX in this line (Figure S4F). Thus, while γ-H2AX may contribute to the radiation-sensitizing effect of AM-101, it appears it may not play a deciding role. 

## 4. Discussion

**Figure 7:**
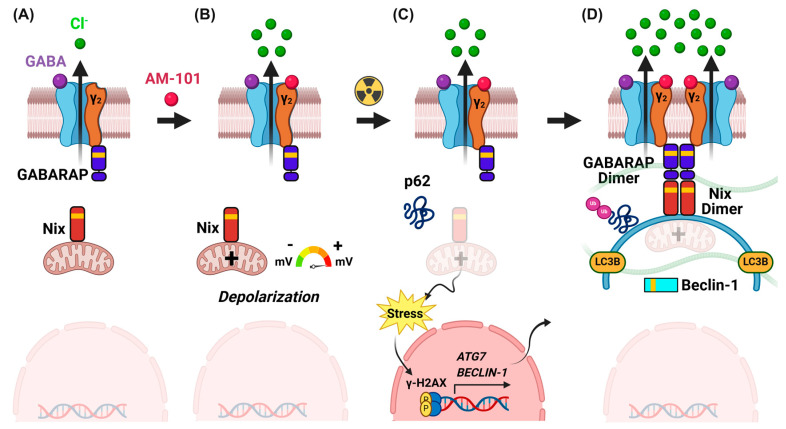
Model of GABA(A)-receptor-mediated autophagy. (**A**) NSCLC cells possess intrinsic GABA(A) receptors (chloride anion channels). (**B**) AM-101 activates GABA(A) receptors, which in turn depolarize mitochondria. Changes in the cancer cell by binding of AM-101 to the receptor in combination with radiation include (i) enhanced expression–abundance of key genes involved in autophagy, including *ATG7* and *BECLIN-1*; (ii) increased phosphorylation of the histone variant H2AX to generate γ-H2AX. (**C**) Depolarization induces key autophagic events in synergy with radiation: (i) enhanced expression and dimerization of GABA(A) receptor-associated protein, GABARAP; (ii) stabilization and dimerization of Nix, coupling GABARAP to mitochondria; (iii) enhanced expression of autophagy-associated proteins Beclin-1 and ATG7; (iv) utilization of ubiquitin-binding protein p62. Nix dimerization increases its stability and coordinates the nucleation of autophagosome formation. In this manner, GABA(A) receptor activation induces complex multimerization, activating autophagy. (**D**) Over time, GABARAP multimerizes commensurate with multimerization of the GABA(A) receptor, which enhances its activity. Created with BioRender.com.

GABA(A) receptors are the major inhibitory neurotransmitter receptors in primates. GABA(A) receptors also have roles outside of the CNS, and receptor subunits are expressed in cancer cells [19,20]. We found in lung adenocarcinoma cells that GABA(A) receptors are functional and that their activation enhances the effect of its natural agonist and metabolite, GABA (Figure 1D). Previously, we found that the activation of GABA(A) receptors in cancer cells contributes to an efflux of chloride anions [21]. Previously, and in this study, we found that GABA(A) receptor activation leads to the depolarization of the mitochondrial transmembrane, consistent with a net efflux of chloride anions [21]. Depolarization has been reported to lead to the induction of autophagy [65]. We therefore investigated changes in levels of proteins with disparate roles in autophagy, including ATG7, the conjugate ATG12-ATG5, and Beclin-1, and found changes in their protein abundance. We analyzed the levels of autophagy substrate protein p62 and found that GABA(A) receptor activation reduces p62 protein levels, indicating the utilization of the autophagy substrate. We were particularly drawn to the contribution of GABARAP and Nix as proteins key to the nucleation of autophagosome assembly and bridging the extracellular plasma membrane and the mitochondrial transmembrane. The GABARAP subfamily of proteins promotes autophagy by regulating the activity of kinase ULK1, whose function stabilizes autophagosome formation [66]. In addition, phosphorylated GABARAP traffics GABA(A) receptors to the extracellular plasma membrane, binding to its γ2-subunit [67,68]. Nix is an autophagy receptor that rests in the mitochondrial transmembrane and complexes with GABARAP to recruit mitochondria to autophagosomes [29]. As well as enhancing the expression of GABRARAP and Nix, we found that GABA(A) receptor activation contributes to their multimerization, thus augmenting the autophagic response. GABARAP was shown previously to increase the cell-surface expression of GABA(A) receptors [69]. Amino acid substitutions of key transmembrane residues of Nix (BNIP3L), such as BNIP3LG204A or BNIP3LG208V, disrupt Nix dimer formation, which results in reduced recognition by LC3A-Nix and, consequently, decreased autophagy induction [53]. This particularly supports our conclusion that GABA(A)-receptor-activation-induced Nix dimerization and increased puncta formation play a role in triggering autophagy induction. We hypothesize and illustrate in our model that the multimerization of GABARAP occurs commensurate with the multimerization of GABA(A) receptors and that this macromolecular assembly assumes a cytotoxic state as it drives a significant efflux of chloride anions. This overall concept is consistent with experimental analysis and theoretical modeling, which shows that the multimerization of GABA(A) receptors and, in turn, GABARAP creates a strong localized distribution of charge and enhanced receptor activity [26,27,28]. This would also occur and be commensurate with a stabilization and multimerization of Nix, as we observe (Figure 7). Our proposed model (Figure 7) also illustrates the sequence of events, where p62 utilization and ATG7 upregulation occur a step earlier than GABARAP and Nix dimerization.

In this way, the perturbation of ion homeostasis as sensed and regulated by GABA(A) receptors would lead to the assembly of an autophagosome and potential enclosure and the subsequent recycling of mitochondria. In our previous study employing a syngeneic melanoma model, we also found that AM-101 enhances the infiltration of CD8+ T cells [22].

Interestingly, autophagy plays an important role in regulating the radiation sensitivity of disparate cancer types. In lung cancer, for example, the enhanced expression of Beclin-1 overcomes radiation resistance [41,70], and the induction of autophagy sensitizes NSCLC and glioblastoma cells to radiation [35,71]. Our finding that AM-101 increases Beclin-1 expression and reduces the autophagy substrate p62 in NSCLC cells and tumors supports the idea that the AM-101-mediated enhancement of autophagy by activating the GABA(A) receptor underpins the radiation sensitization of tumors.

## 5. Conclusions

GABA(A) receptors have been a key pharmacologic target to remediate neurological disorders for over seventy years. In this study, we demonstrate that activating GABA(A) receptors can be leveraged to effectively treat primary non-small cell lung cancer (NSCLC) and its brain metastases when used in combination with radiation. The discovery that the GABA(A) receptor activator AM-101 enhances autophagy and sensitizes non-small cell lung cancer (NSCLC) cells to radiation supports its potential use as a therapeutic agent in combination with radiation therapy for both primary and brain-metastatic NSCLC. Moreover, AM-101 is a non-toxic, brain-penetrant radiation sensitizer that can improve tumor control and allow for radiation dose de-intensification to reduce toxicity. 

## Data Availability

Data supporting the findings of this study are available within the article and its Supplementary Information.

## Supplementary Materials

The following supporting information can be downloaded at https://www.mdpi.com/article/10.3390/cancers16183167/s1. Figure S1: GABA(A) receptor subunit gene expression; Figure S2: Effect of AM-101 and diazepam on survival of non-small cell lung cancer cells and primary lung bronchial epithelial cells; Figure S3: Human-relevant ex vivo ‘chip’; Figure S4: Effect of AM-101 and radiation on clonogenicity, Caspase 3 cleavage, and γ-H2AX induction in human primary and patient-derived brain-metastatic lung adenocarcinoma cells; Figure S5: Heterotopic and intracranial orthotopic mouse xenograft experiments with primary and brain-metastatic cell lines; Figure S6: Change in abundance of autophagy biomarkers in tumors in response to GABA(A) activation; Figure S7: Inhibition of AM-101 autophagy by bafilomycin A1 and the mechanism of action of GABARAP binding stapled peptide Pen3-ortho; Figure S8: Full-size blots of Figure 1, panel B; Figure S9: Full-size blots of Figure 4, panels C, D, E; Figure S10: Full-size blots of Figure 5, panels A, B, C, D; Figure S11: Full-size blots of Figure 6, panels B and C; Figure S12: Full-size blots of Figure S4, panels C, D, E; Figure S13: Full-size blots of Figure S6, panels A, B, C, D; Figure S14: Full size blots of Figure S7, panels B and C; Video S1: Videography of mouse intracranial experiments. References [72,73,74] are cited in the supplementary materials.
