# Supplementary material for: GABA(A) Receptor Activation Drives GABARAP–Nix Mediated Autophagy to Radiation-Sensitize Primary and Brain-Metastatic Lung Adenocarcinoma Tumors
Source: Cancers (Basel). 2024 Sep 15;16(18):3167. doi: 10.3390/cancers16183167 (PMC11430345; doi:10.3390/cancers16183167)
Supplement: Supplementary file 1 [file cancers-16-03167-s001.zip › cancers-3094271-supplementary-2nd.pdf]

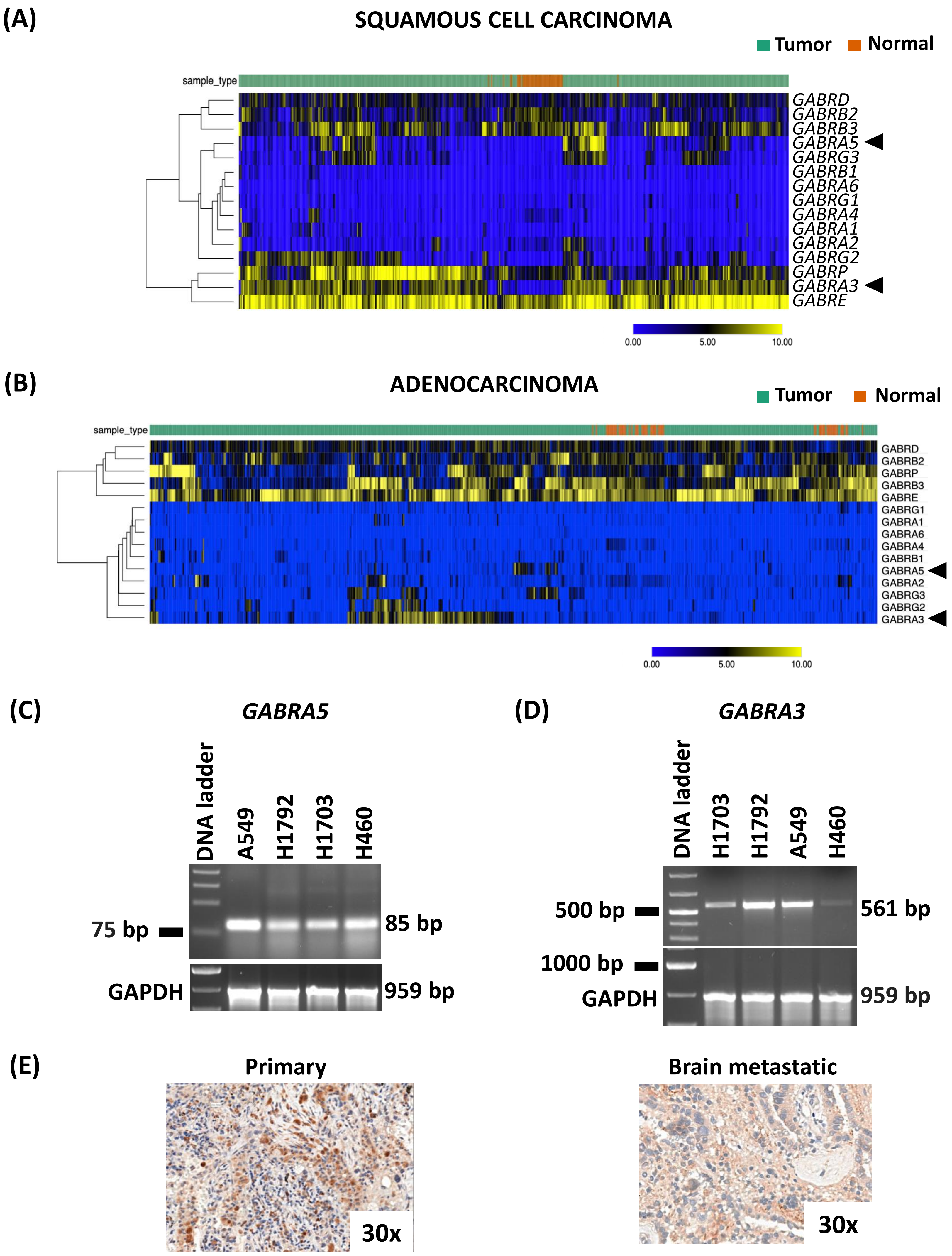

Figure S1. GABA(A) receptor subunit gene expression. Heatmaps of GABA(A) receptor mRNA expression analysis from RNA-seq datasets of patient samples from pre-processed TCGA lung squamous cell carcinoma (A) and adenocarcinoma (B) primary tumors. The pre-processed TCGA lung squamous cell carcinoma (SC)[1] and lung adenocarcinoma (AC)[2] RNA-seq datasets were downloaded from the integrative LINCS (iLINCS) portal[3] and filtered to remove metastatic and recurring tumor samples. The expression profiles of GABA(A) genes were clustered and heatmaps were created using Morpheus (https://software.broadinstitute.org/morpheus/). *GABRA5* (C) and *GABRA3* (D) mRNA levels were assessed by RT-PCR in a panel of human NSCLC cell lines. *GAPDH* mRNA was amplified and used as an endogenous control. RT-PCR for GABRA (E) IHC of GABRA3 stained sections of human primary lung adenocarcinoma tissue section (left) and patient matched tissue section of lung brain metastatic adenocarcinoma (right). Patient tumor tissue was obtained from the CLIA certified University of Cincinnati Histopathology Core Laboratory under an approved IRB.

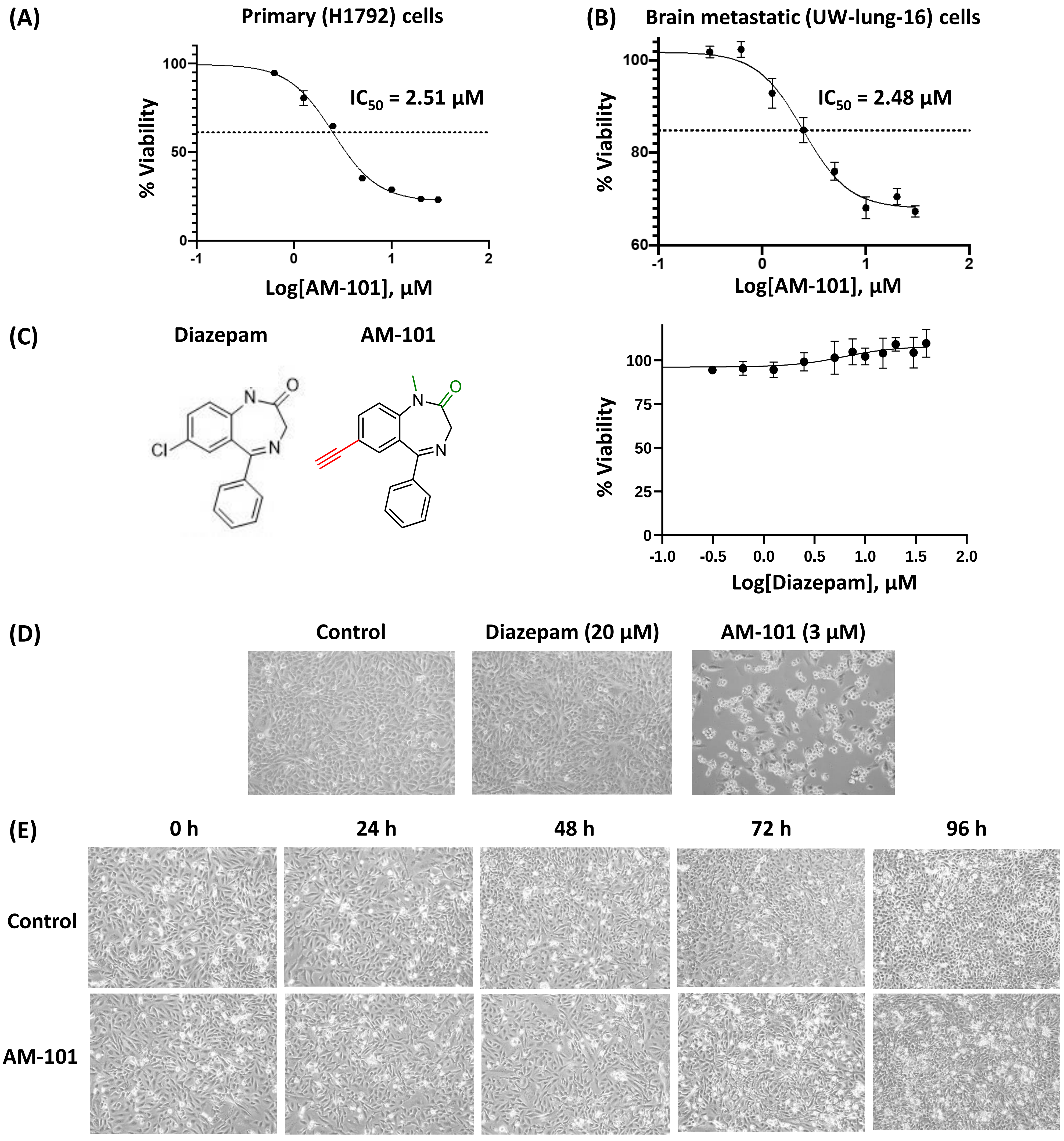

Figure S2. Effect of AM-101 and diazepam on survival of non-small cell lung cancer cells and effect of AM-101 on primary lung bronchial epithelial cells. Log transformed curves from MTS assay of AM-101 incubated with lung adenocarcinoma (H1792) (A) and lung brain metastatic (UW-lung-16-GFP-Luc) cells (B). (C) Chemical structures of diazepam (left) and AM-101 (middle) showing the ethinyl bond in place of a chloride at position 7 in the 1,4-benzodiazepine ring system. Log transformed curves from *in vitro* MTS assay with diazepam on H1792 cells (right). Due to lack of a cytotoxic effect, an IC_{50} value is not reported. (D) Effect of treatment of Control (DMSO), diazepam (20 µM), or AM-101 (3 µM) on H1792 cell viability, as analyzed by brightfield microscopy. An equal number (~0.6 x 10^6) of H1792 cells were grown overnight. When the cells adhered to the surface of the culture plate, cells were treated for 48 hrs with Control, diazepam, or AM-101 (n=2 per treatment group). After 48 hr, cells were imaged in brightfield under a Leica DMi1 inverted microscope at 10X magnification. (E) Brightfield microscopic images of a time course study to determine the effect of AM-101 treatment on BEAS-2B cells (normal human lung bronchial epithelial cells). Equal number of cells were plated in two wells of a 6-well plate. Once the cells reached a steady growth phase and the culture reached 70% confluency, one set of cells was treated with AM-101 (2.5 µM) dissolved in DMSO, while the cells in Control group were treated with DMSO (the diluent) in identical volumes. Both groups of cells were analyzed by brightfield microscopic images captured at indicated timepoints using a Leica DMi1 inverted microscope with a camera at 10X magnification.

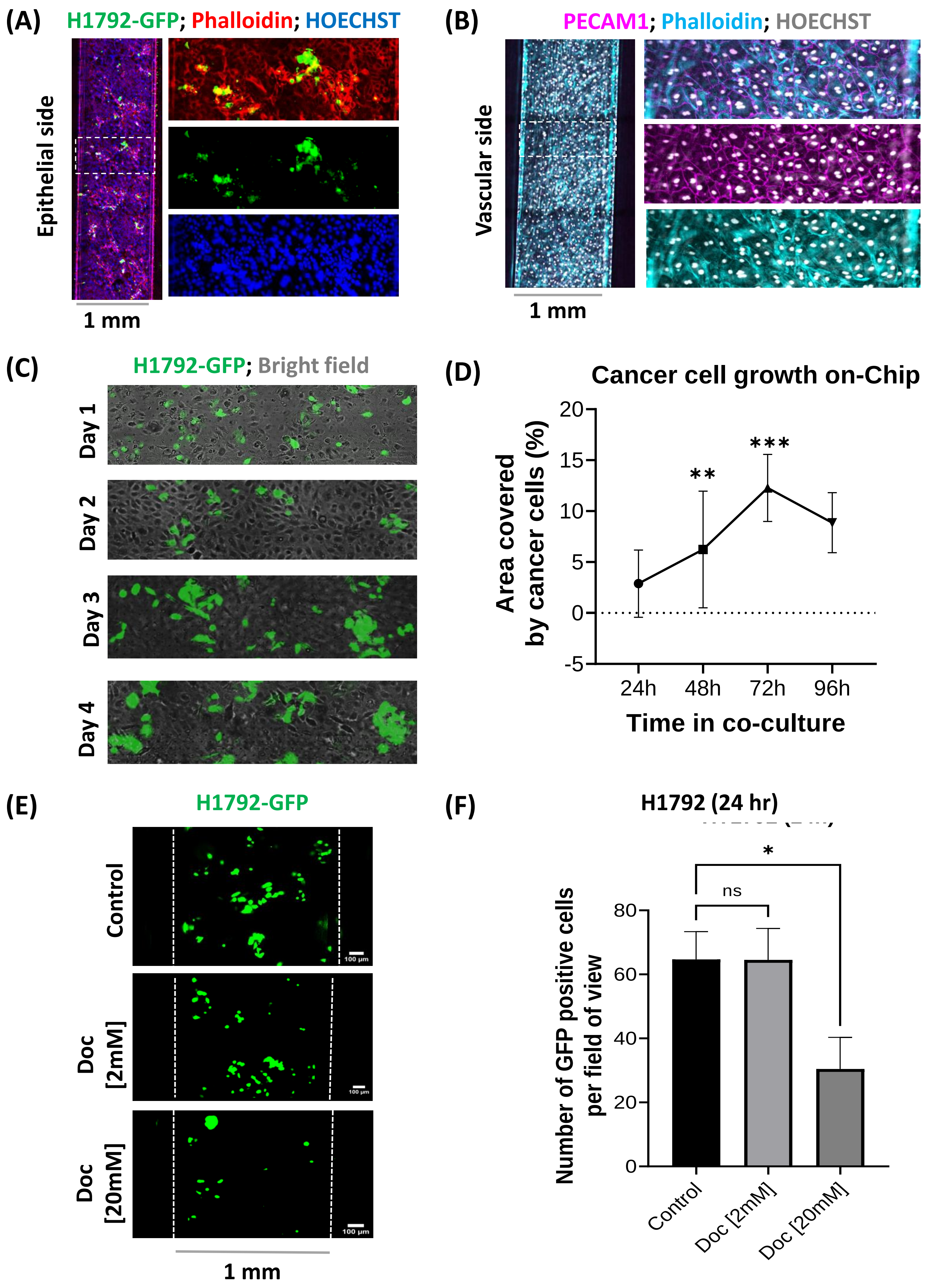

Figure S3. Human-relevant *ex vivo* 'chip'. (A) Immunofluorescent staining of the Lung-on-Chip's epithelial compartment co-cultured with H1792-GFP cells (green) reveals actin filaments (red, phalloidin) and nuclei (blue, HOECHST) localization. (B) The vascular compartment exhibits staining for PECAM1 (magenta, endothelial cell marker), actin filaments (cyan, phalloidin), and nuclei (grey, HOECHST). (C) H1792-GFP cells grow and spread through the epithelial compartment of the Lung-on-Chip over time. (D) Results of image analysis and quantitation of the GFP signal spread over time expressed as a percentage of area covered by the GFP signal. (E) H1792-GFP cells undergo cell death in response to docetaxel, leading to (F) a reduction in the number of GFP-positive cells. The estimated number of GFP-positive cells was assessed using background subtraction and signal thresholding, followed by particle analysis using a Fiji plugin to estimate the number of GFP+ cells per field of view in each testing condition. The choice of particle analysis over a percentage of the area covered by the signal was based on its ability to provide discrete values, resulting in a more sensitive measurement.

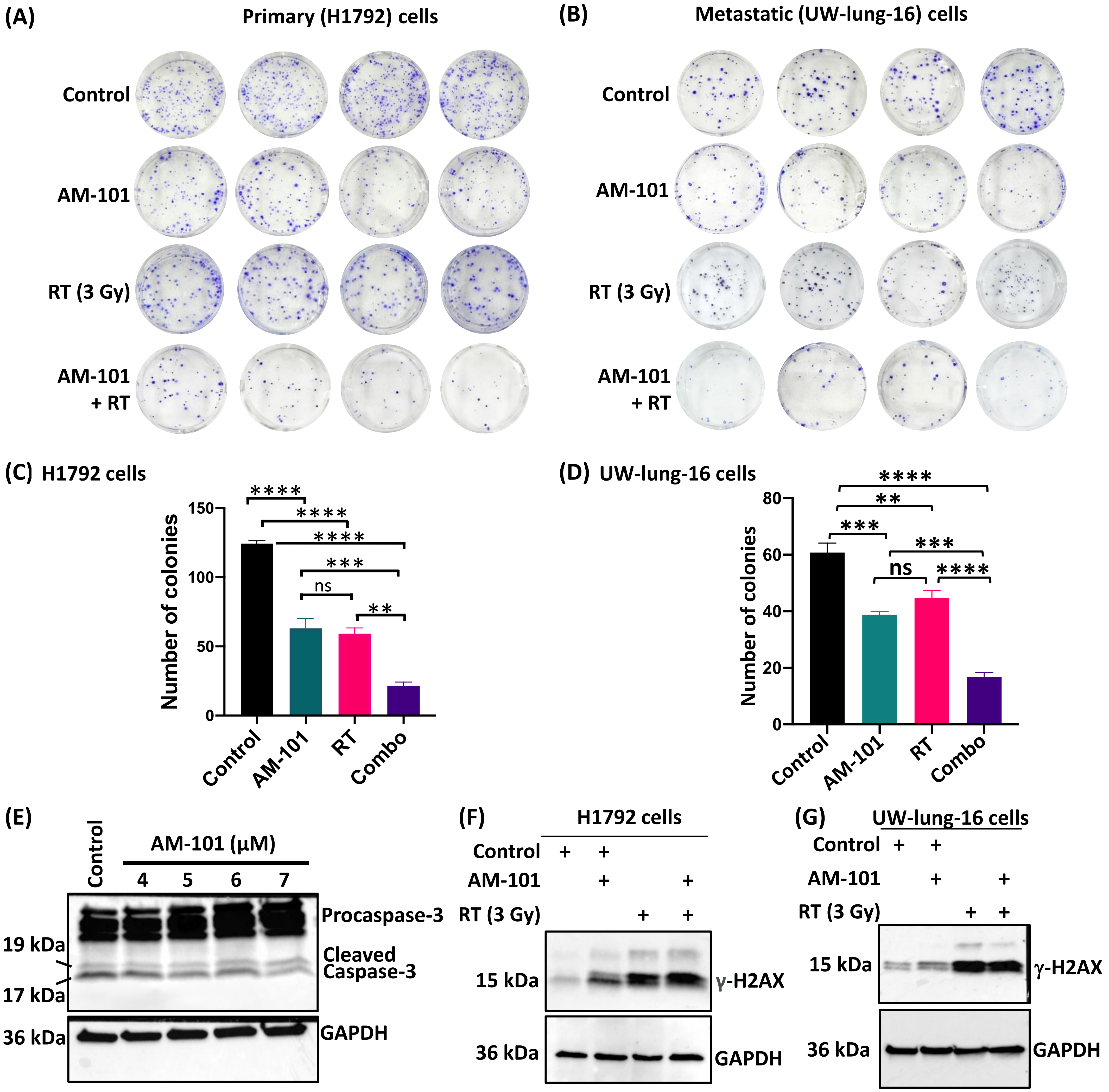

Figure S4. Effect of AM-101 and radiation on clonogenicity, Caspase 3 cleavage, and γ-H2AX induction in human primary and patient derived brain metastatic lung adenocarcinoma cells. (A) Effect of AM-101 and radiation on clonogenicity of lung adenocarcinoma (H1792) cells. Control is DMSO treated and unirradiated cells. RT: Radiation therapy (3 Gy). AM-101: 2.5 μM. (B) Effect of AM-101 and radiation on clonogenicity of patient derived brain metastatic UW-lung-16 cells. RT: Radiation treatment (3 Gy). AM-101: 2.5 μM. For both (A) and (B) colonies containing more than 40 individual cells were counted under the microscope. (C) Bar graph showing the quantification of stained colonies for each experimental group in the clonogenic assay with H1792 cells. One-way ANOVA was performed with Tukey's multiple comparisons test, $p > 0.05$ (ns), $0.001 < p < 0.01$ (**), $0.0001 < p < 0.001$ (***), $p < 0.0001$ (****). Combo: AM-101 + RT (3 Gy). N = 4 biological replicates per group, mean ± SEM. (D) Bar graph showing the quantification of stained colonies for each experimental group in the clonogenic assay with UW-lung-16 cells. One-way ANOVA was performed with Tukey's multiple comparisons test. Control vs AM-101, *** p = 0.0001; Control vs RT ** p = 0.0019; Control vs Combo **** p < 0.0001; AM-101 vs RT, ns (not significant), p = 0.308; AM-101 vs Combo *** p = 0.0001; RT vs Combo **** p < 0.0001. N = 4 biological replicates per group, mean ± SEM. (E) Effect of AM-101 treatment on Caspase-3 cleavage in H1792 (primary lung adenocarcinoma) cells was analyzed by immunoblotting. H1792 cells were exposed to multiple concentrations of AM-101 (ranging from 4 to 7 μM), all exceeding the IC_{50} value of AM-101, for a duration of 48 hrs. Following treatment, cell lysates were used for immunoblotting by probing with a caspase-3 antibody (Cell Signaling). DMSO treated cells served as the Control since DMSO is the diluent of AM-101. GAPDH is used as a loading control. (F) Immunoblotting of DNA damage marker γ-H2AX protein with lysates of lung adenocarcinoma H1792 cells treated with radiation (3 Gy) or AM-101 (3 μM) or a combination of both. (G) Immunoblotting of γ-H2AX protein with lysates of brain metastatic UW-lung-16 cells treated with radiation (3 Gy) or AM-101 (3 μM) or a combination of both. For both experiments in E and F, cell lysates were collected 20 hr after treatment. GAPDH is used as a loading control.

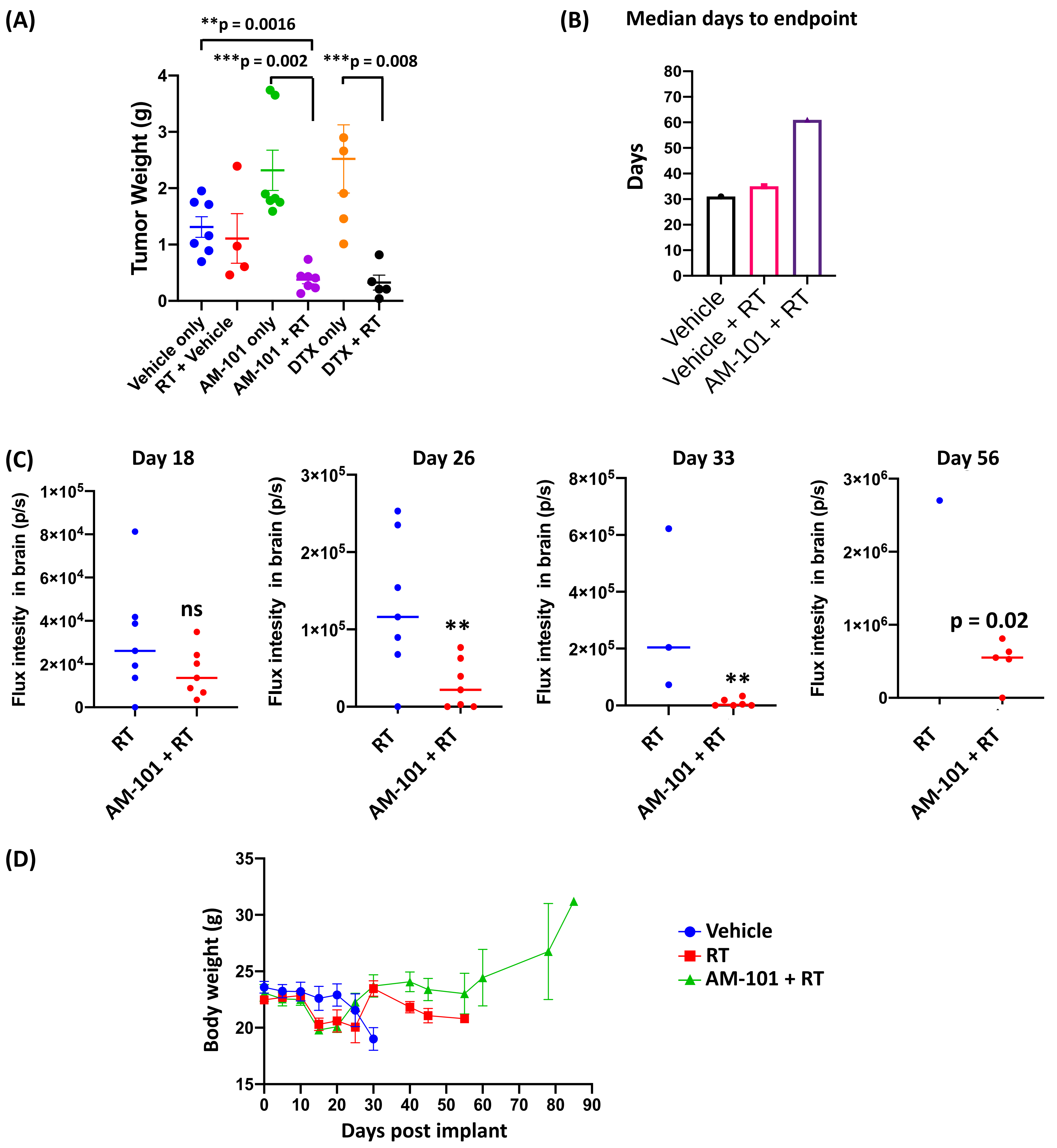

Figure S5. Heterotopic and intracranial orthotopic mouse xenograft experiments with primary and brain metastatic cell lines. (A) Weights of excised subcutaneous H1792 xenograft tumors in mice from different treatment groups at their experimental endpoints are represented in scatterplot graphs. Data represented as mean ± S.E. Mice were euthanized at experimental endpoints, and subcutaneous xenograft tumors were excised weighed, and preserved at appropriate temperature for protein expression studies. Student's t-test was performed to compare the means of two respective groups and to adjust for the multiplicity Bonferroni's correction was used. The p-values between each pair of groups compared are indicated. Due to the Bonferroni correction and as we did 4 different comparisons a value of $p < 0.0125$ is considered significant. (B) Bar graphs showing median days to the endpoint in different treatment groups of mice with UW-lung-16 intracranial orthotopic xenograft tumors. (C) Flux intensities of UW-lung-16 implanted brain metastatic tumors at different time points of luciferase imaging in mice treated with radiation or radiation and drug (AM-101). (D) Mean body weights of UW-lung-16 intracranial brain metastatic tumor-bearing mice from three groups receiving different treatments. The body weight of mice was recorded over time during and after treatment.

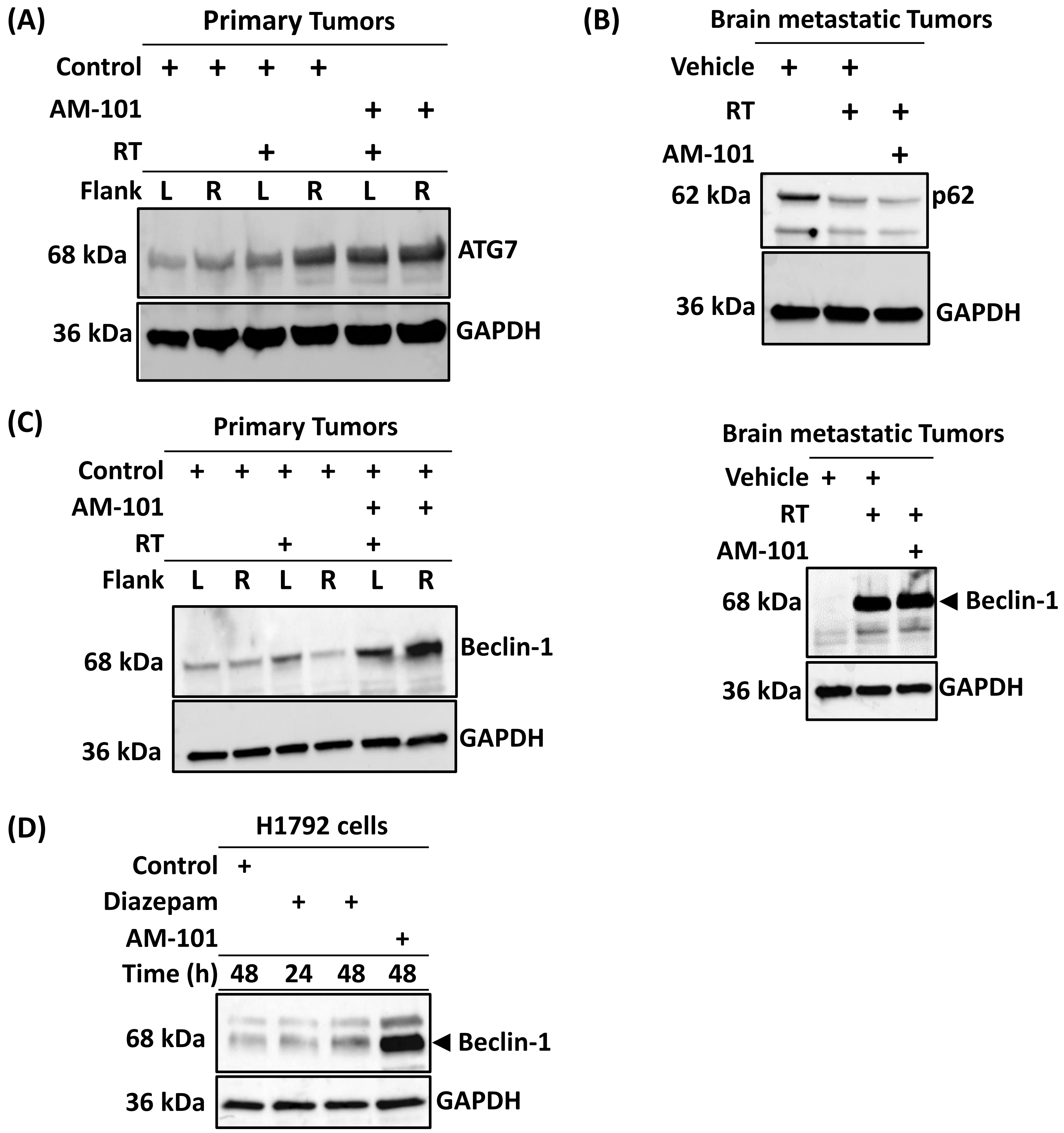

Figure S6. Change in abundance of autophagy biomarkers in tumors in response to GABA(A) activation. (A) Immunoblotting of ATG-7 protein in lysates from subcutaneous H1792 xenograft tumors in NSG mice, comparing treatment groups: Control (Vehicle), radiation (RT), AM-101, and combination of RT and AM-101. RT: radiation therapy (dose: 5 Gy), L: left flank tumor; R: Right flank tumor. (B) Immunoblotting of intracranial xenograft tumor tissue lysate. Radiation treatment (RT) shows reduced level of p62. Combination treatment with RT plus AM-101 shows a greater utilization of p62, based on the band intensity. GAPDH is used as a loading control. (C) Beclin-1 immunoblots of lung adenocarcinoma primary (H1792) and brain metastatic (UW-lung-16) tumors following treatment with AM-101 ± radiation therapy (RT). Left, Beclin-1 protein expression is enhanced in H1792 tumors by RT and AM-101 alone. Combined treatment does not appear to enhance Beclin-1 in tumors relative to AM-101 alone. Expression of Beclin-1 protein evaluated by immunoblotting of lysates from H1792 subcutaneous xenograft tumors in NOD scid gamma (NSG) mice from different experimental groups receiving Vehicle (Control) with or without radiation or AM-101 or radiation (RT) alone or combination of radiation and AM-101. Radiation (RT) dose: 5 Gy, L: left flank tumor; R: right flank tumor. Control, vehicle-treated. Right, Immunoblotting to detect Beclin-1 expression in intracranial UW-lung-16 xenograft tumor tissue of athymic nude mice harvested from 3 treatment groups receiving vehicle, or radiation plus vehicle, and radiation plus AM-101. (D) Immunoblotting was performed to assess the effects of AM-101 and diazepam, a benzodiazepine, on Beclin-1 protein levels in H1792 cells. The control group received vehicle treatment. H1792 cells treated with AM-101 (3 µM) were harvested 48 hours post-treatment, while cells treated with diazepam (20 µM) were harvested at 24 and 48 hrs post-treatment. GAPDH was used as a loading control in all immunoblotting experiments.

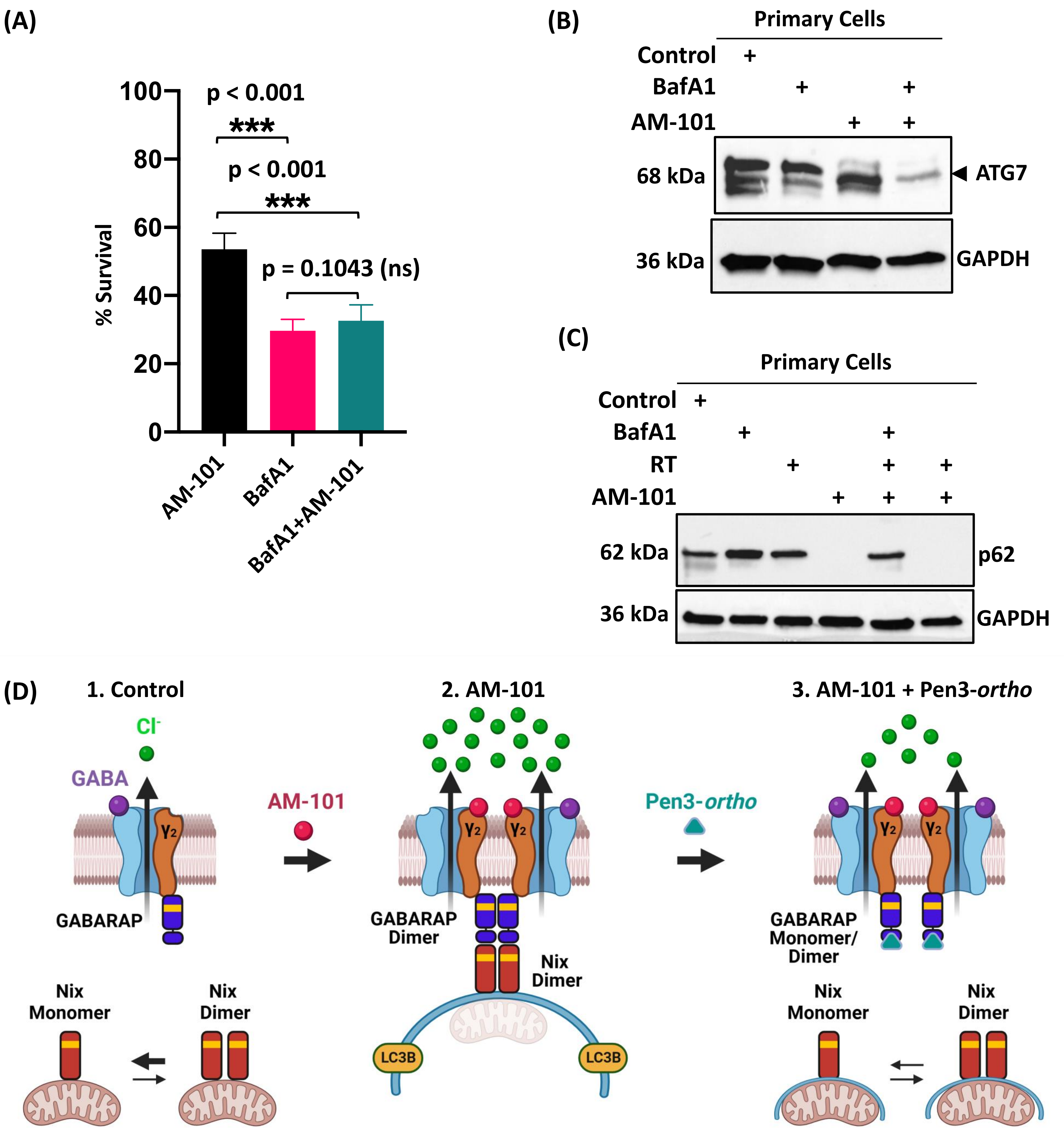

Figure S7. Inhibition of AM-101 autophagy by bafilomycin A1 and the mechanism of action of GABARAP binding stapled peptide Pen3-*ortho*. (A) Bar graphs showing the effect of combining the autophagy inhibitor bafilomycin A1 (10 nM for 5 hours) with AM-101 on the survival of human H1792 NSCLC cells, measured by an MTS assay 48 hours post-treatment. Student's t test was performed to assess the significance of difference between each pair of groups (B) Changes in ATG7 protein expression were observed when H1792 cells were treated with AM-101, bafilomycin A1, or both (C). p62 and GAPDH were used as loading controls. Changes in expression of p62 protein as assessed by immunoblotting of lysates from H1792 cells treated with AM-101, bafilomycin A1 (50 nM), radiation (3 Gy) and triple combination of bafilomycin A1, AM-101 plus radiation (RT) and a combination of radiation (RT) plus AM-101 only. Control, DMSO. (D) Model showing how Pen3-*ortho* inhibits the cytotoxic effect of AM-101. Pen3-*ortho* binds GABARAP in the same pocket where Nix associates with GABARAP, thus acting as a competitive inhibitor. (1) Control, H1792 cells, vehicle treated. (2) AM-101 treatment promotes GABARAP and Nix interaction which triggers autophagosome membrane formation leading to autophagy. (3) Pen3-ortho, when used with AM-101, competitively inhibits the binding of GABARAP to Nix. This reduces the levels of Nix monomers and dimers, thereby blocking the pro-autophagy effect of AM-101.

Figure S8: Full size blots of Figure 1, panel B.

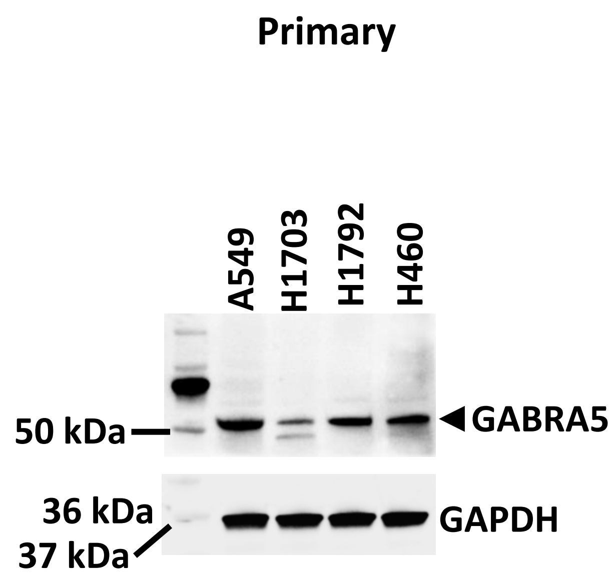

The blot was cut into two strips to probe with GABRA5 and GAPDH antibodies separately.

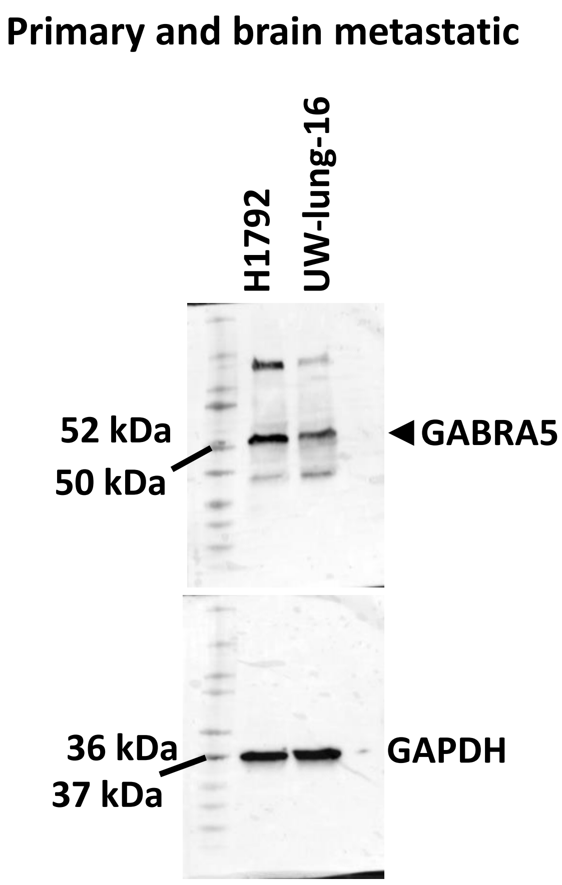

Figure S9: Full size blots of Figure 4, panels C, D, E

(4C)

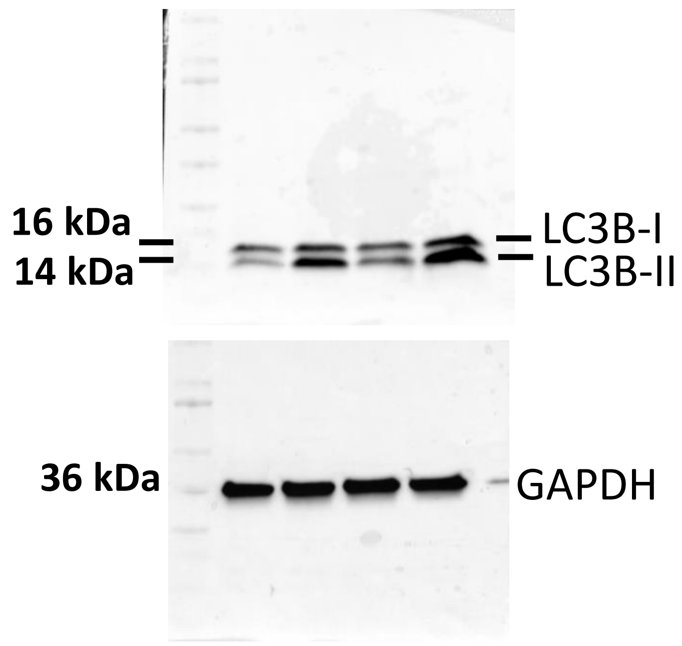

(4D) **GABARAP (primary)**

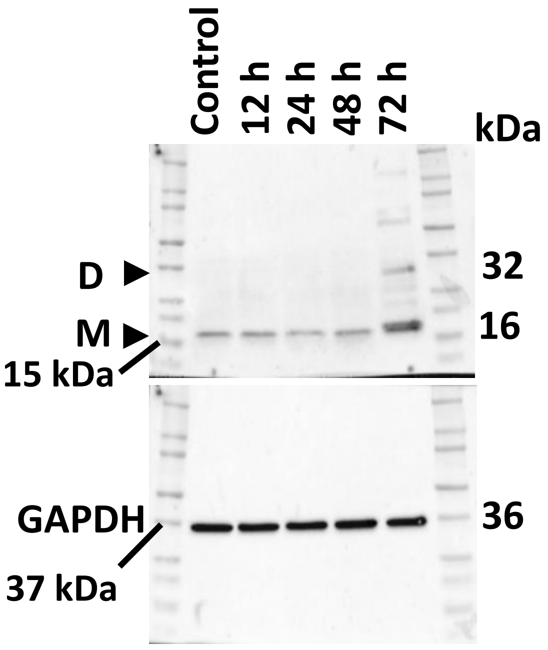

D: dimer. M: monomer

GABARAP (brain metastatic)

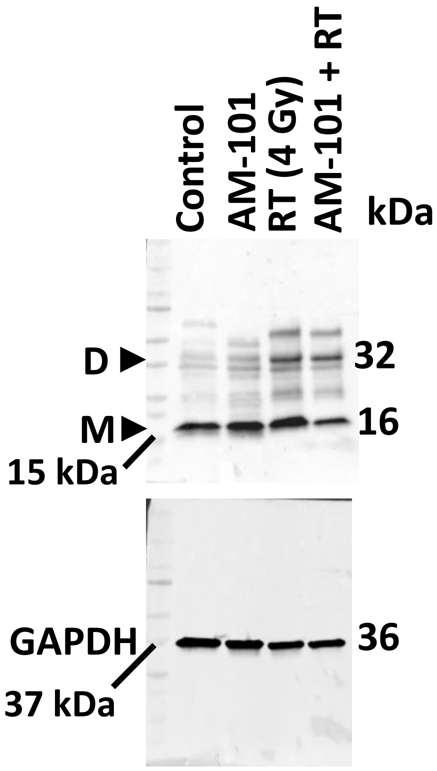

D: dimer. M: monomer

(4E) **Nix (primary)**

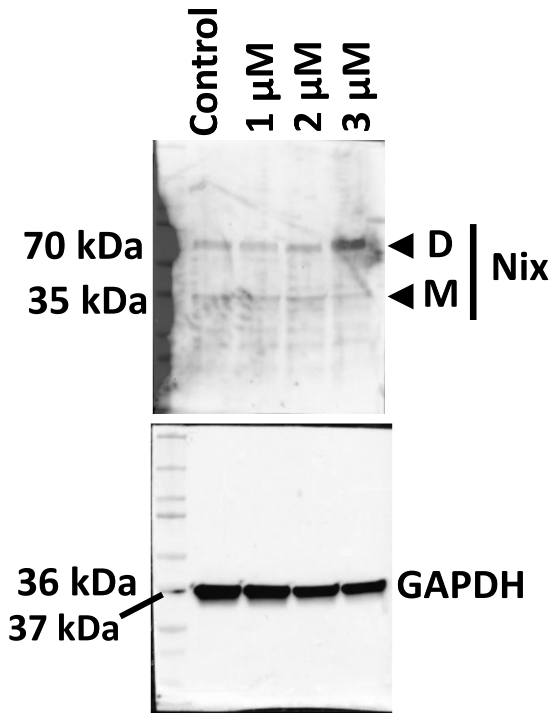

Figure S10: Full size blots of Figure 5, panels A, B, C, D.

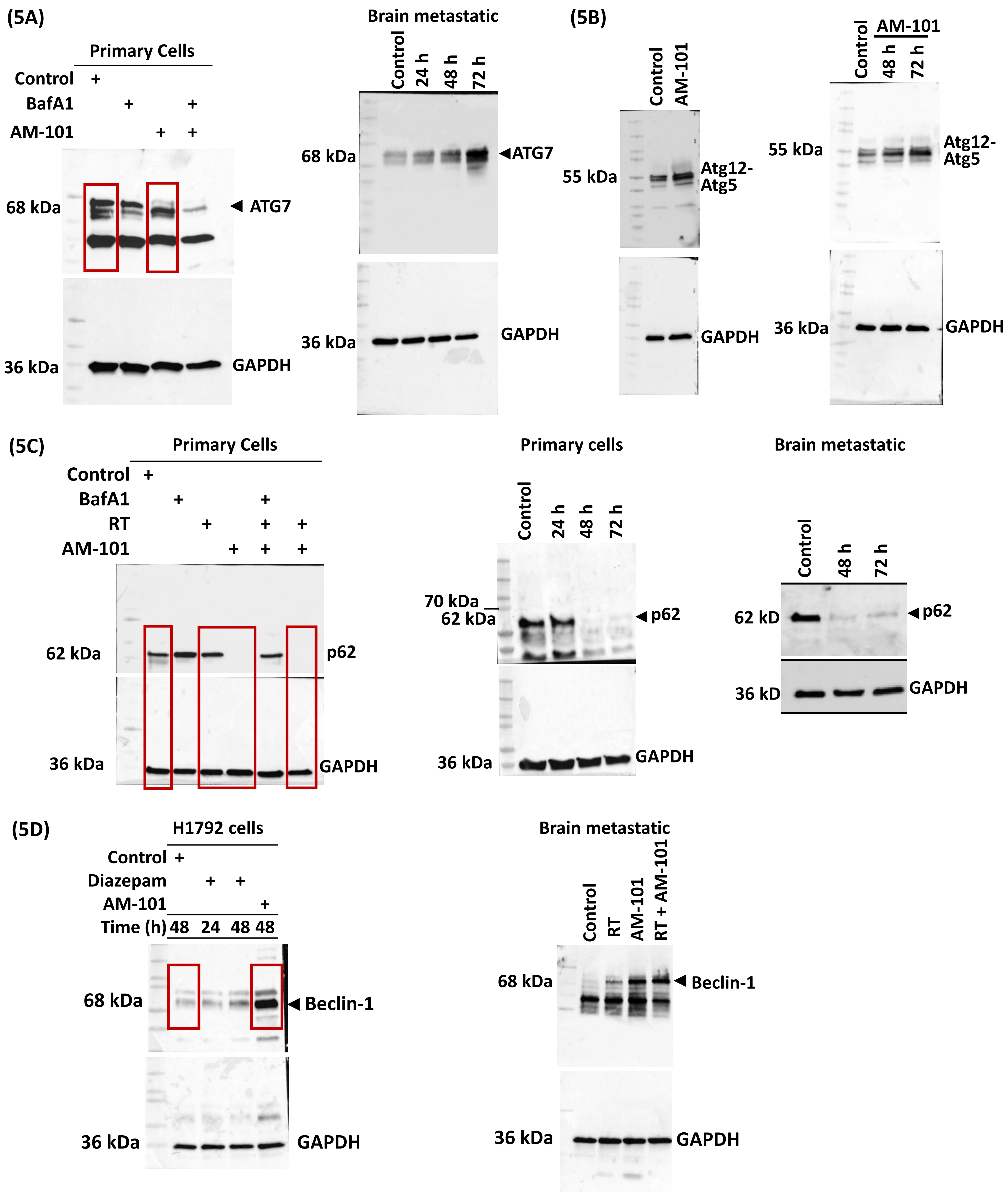

Figure S11: Full size blots of Figure 6, panels B, C.

(6B)

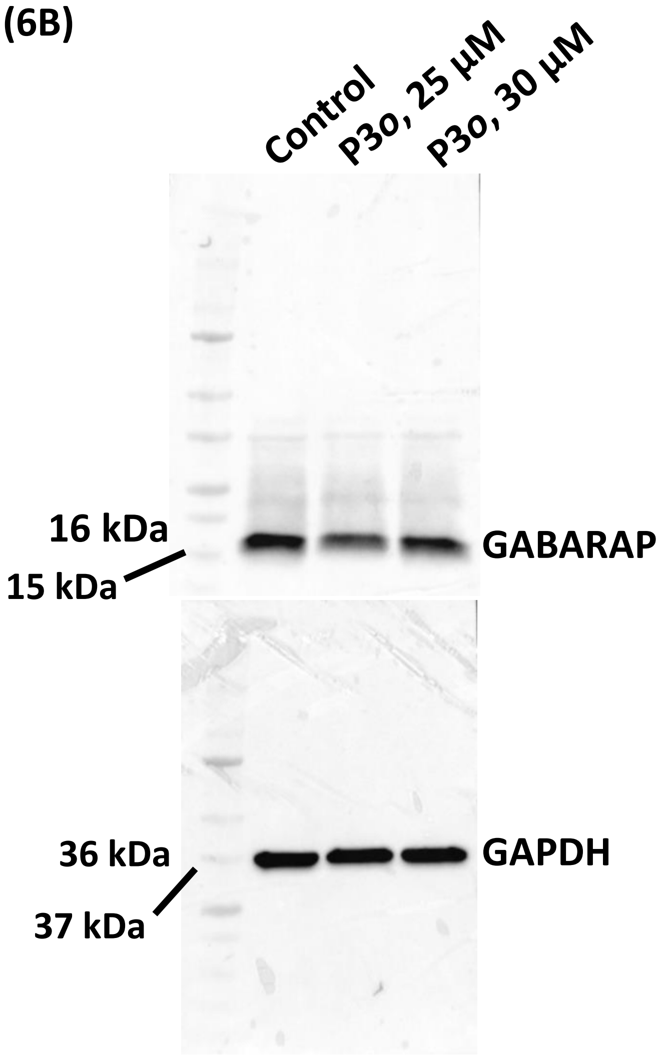

(6C)

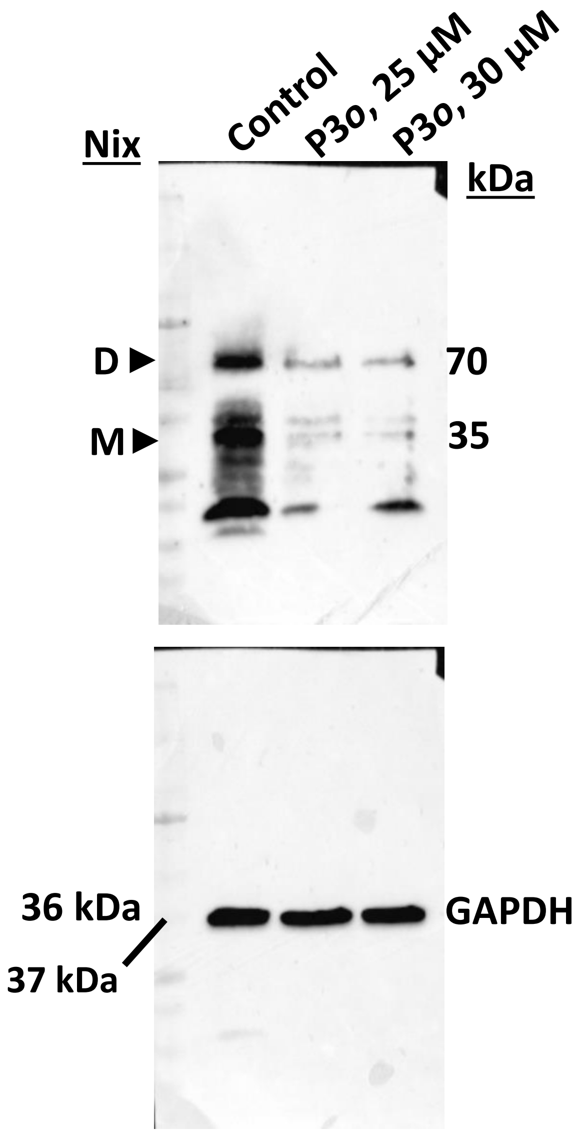

Figure S12: Full size blots of Figure S4, panels C, D, E.

(S4C)

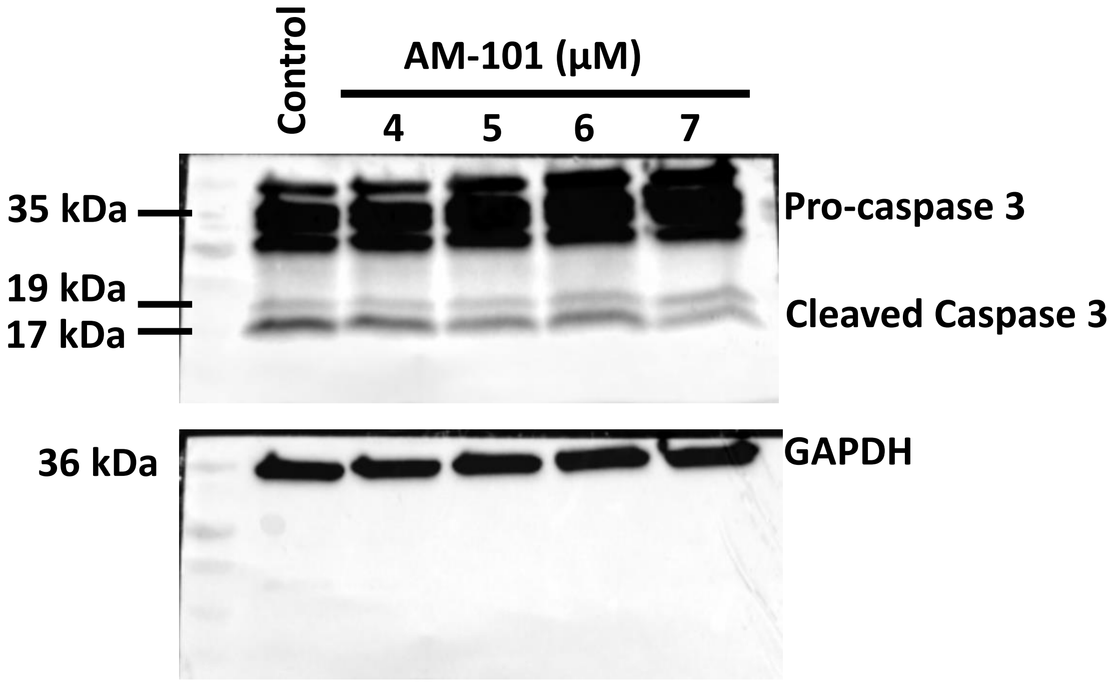

(S4D)

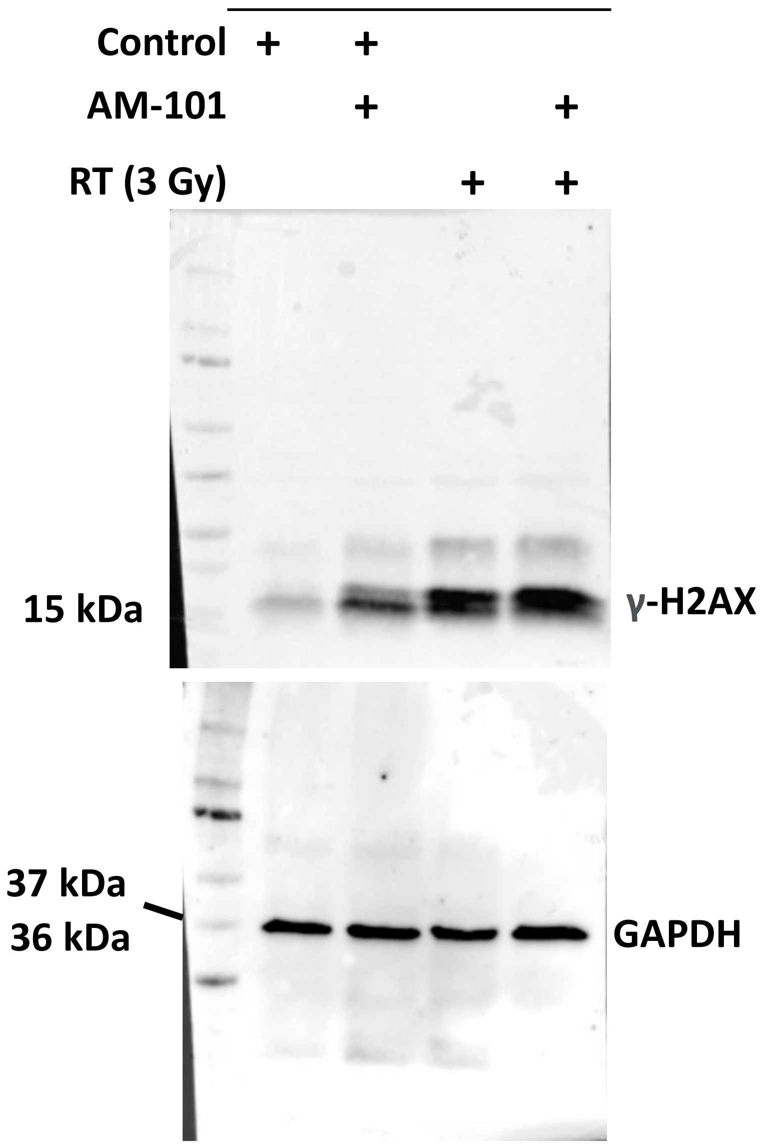

(S4E)

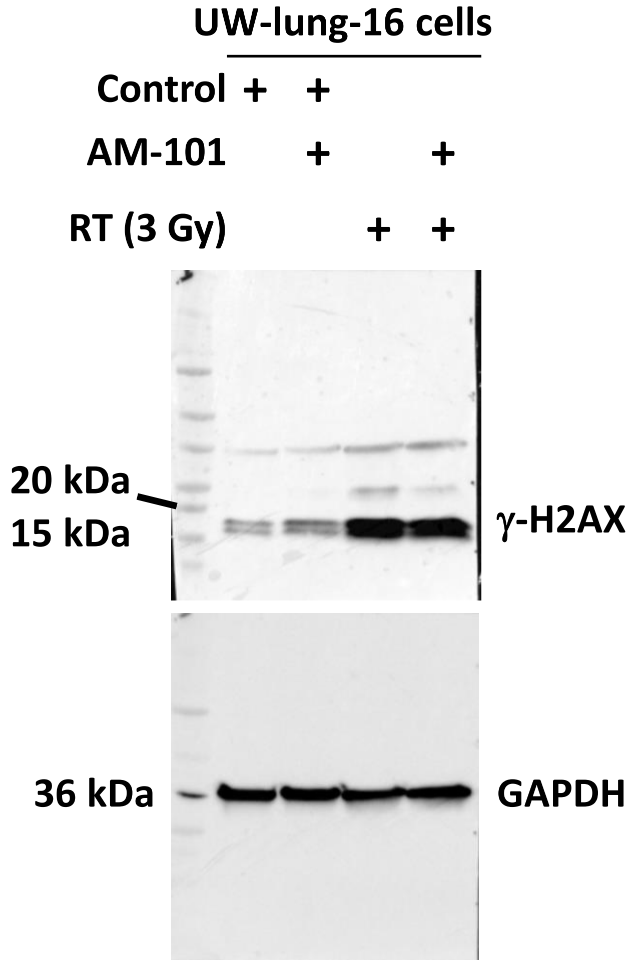

Figure S13: Full size blots of Figure S6, panels A, B, C, D.

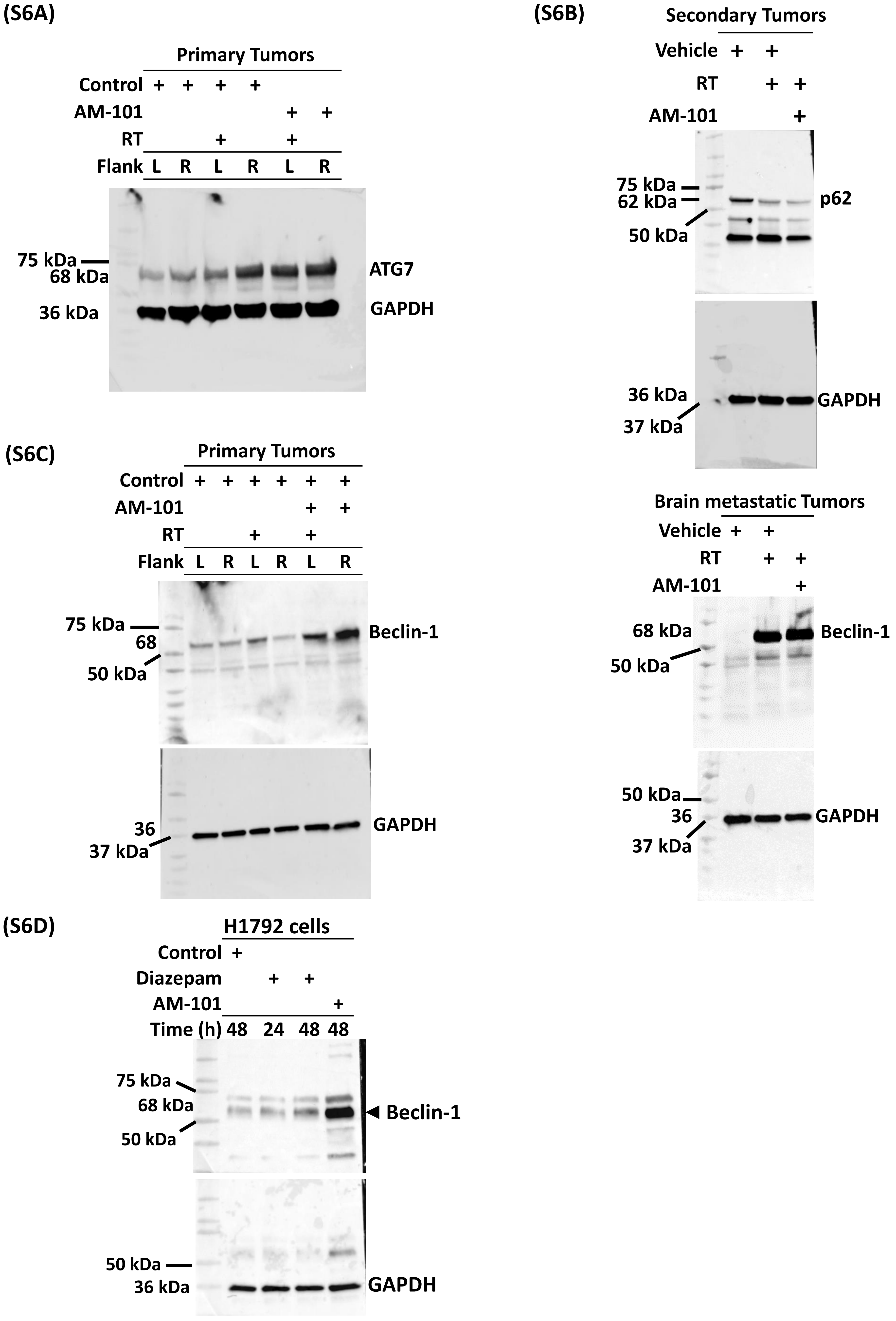

Figure S14: Full size blots of Figure S7, panels B, C.

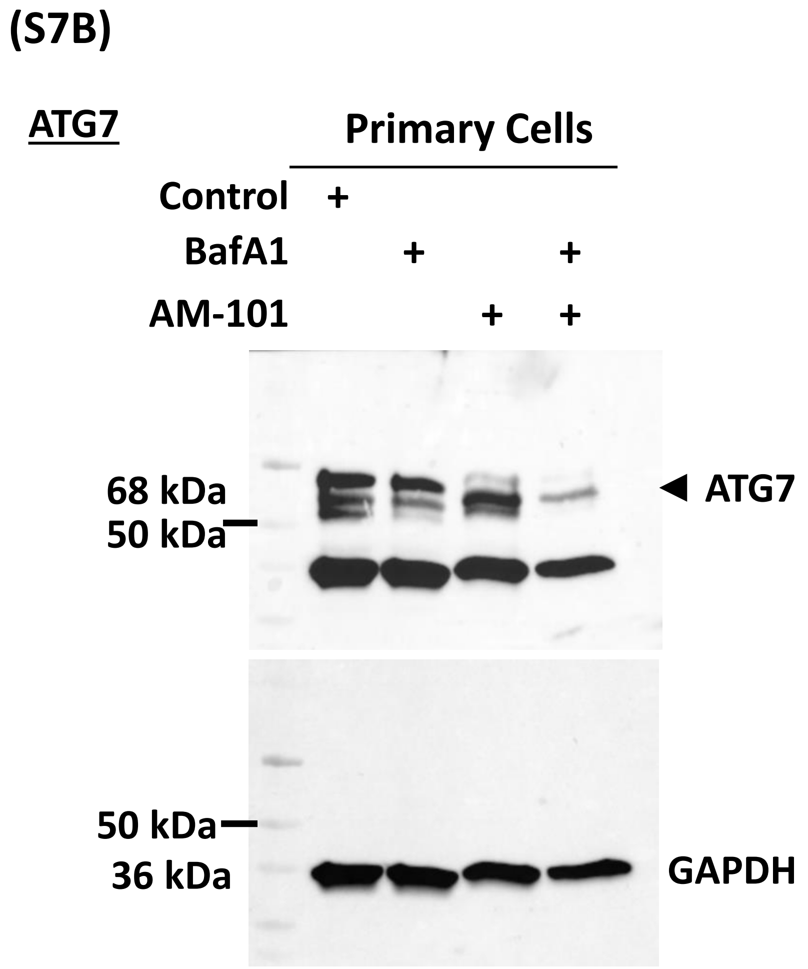

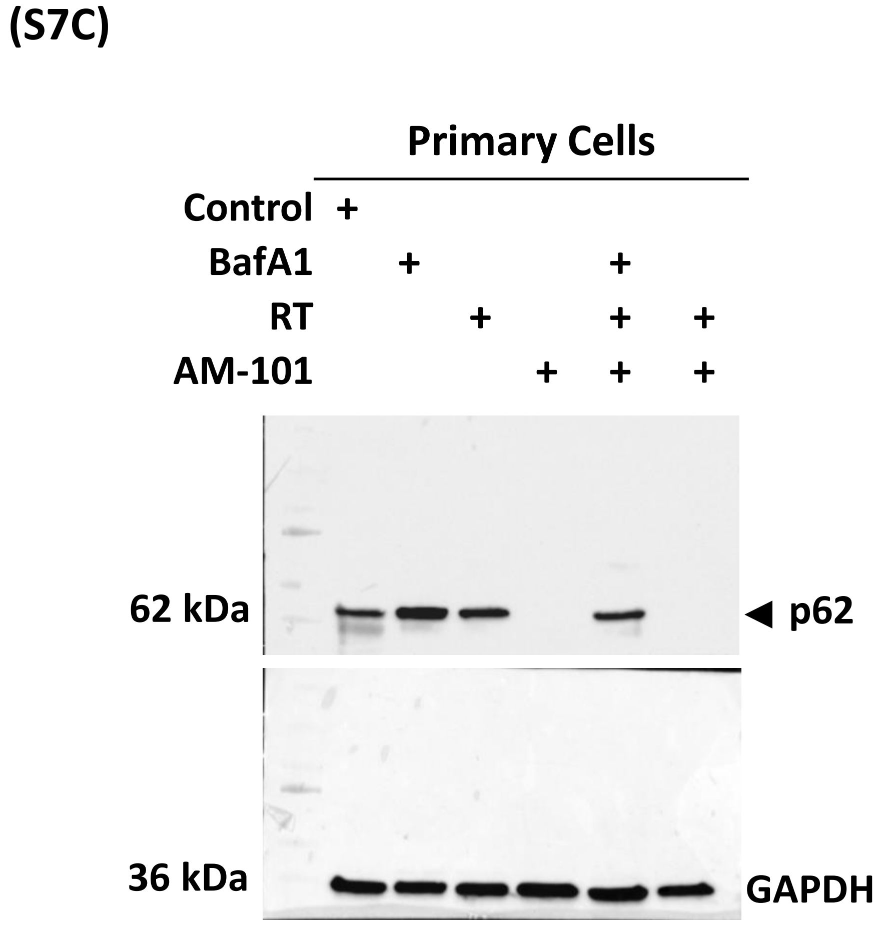

Supplementary References

1. The Cancer Genome Atlas Research Network. Comprehensive genomic characterization of squamous cell lung cancers. *Nature,* **2012**, 489(7417):519-525. 10.1038/nature11404.
2. The Cancer Genome Atlas Research Network. Comprehensive molecular profiling of lung adenocarcinoma. *Nature,* **2014**, 511(7511):543-550. 10.1038/nature13385.
3. Pilarczyk, M. et al. Connecting omics signatures and revealing biological mechanisms with iLINCS. *Nat Commun* **2022**, 13(1):4678.10.1038/s41467-022-32205-3.
